# Mapping Dermatology Life Quality Index (DLQI) scores to EQ-5D utility scores using data of patients with atopic dermatitis from the National Health and Wellness Study

**DOI:** 10.1007/s11136-020-02499-1

**Published:** 2020-04-15

**Authors:** Andreas Westh Vilsbøll, Nana Kragh, Julie Hahn-Pedersen, Cathrine Elgaard Jensen

**Affiliations:** 1 LEO Pharma, Ballerup, Denmark; 2 Department of Clinical Medicine, Danish Center for Healthcare Improvements, Aalborg University, Aalborg, Denmark

**Keywords:** Mapping, Dermatology Life Quality Index (DLQI), EuroQoL-5-dimension (EQ-5D), Atopic Dermatitis (AD)

## Abstract

**Purpose:**

To develop a mapping algorithm for generating EQ-5D-5-level (EQ-5D-5L) utility scores from the Dermatology Life Quality Index (DLQI) in patients with atopic dermatitis (AD).

**Methods:**

The algorithm was developed using data from 1232 patients from four countries participating in the National Health and Wellness Study. Spearman’s rank correlation coefficient was used to evaluate the conceptual overlap between DLQI and EQ-5D-5L. Six mapping models (ordinary least squares [OLS], Tobit, three different two-part models, and a regression mixture model) were tested with different specifications to determine model performance and were ranked based on the sum of mean absolute error (MAE), and root mean squared error (RMSE).

**Results:**

The mean DLQI score was 7.23; mean EQ-5D-5L score was 0.78; and there were moderate negative correlations between DLQI and EQ-5D-5L scores (*p* = − 0.514). A regression mixture model with total DLQI, and age and sex as independent variables performed best for mapping DLQI to EQ-5D-5L (RMSE = 0.113; MAE = 0.079).

**Conclusion:**

This was the first study to map DLQI to EQ-5D-5L exclusively in patients with AD. The regression mixture model with total DLQI, and age and sex as independent variables was the best performing model and accurately predicted EQ-5D-5L. The results of this mapping can be used to translate DLQI data from clinical studies to health state utility values in economic evaluations.

**Electronic supplementary material:**

The online version of this article (10.1007/s11136-020-02499-1) contains supplementary material, which is available to authorized users.

## Introduction

Atopic dermatitis (AD) is a chronic, inflammatory skin condition with a wide range of symptoms affecting not only the skin, but also the immune system, sleep, mental health, and quality of life (QoL) [1]. Evidence suggests that AD “is the leading non-fatal health burden attributable to skin diseases” [2]. QoL is measured in less than 30% of AD studies [3], and there is no single instrument recommended to measure health-related QoL (HRQL) in patients with AD [4], with some reviews finding up to 18 different measures used in AD studies [3]. Certain jurisdictions also have certain preferences for instruments, [5, 6] and depending on the jurisdiction and health technology assessment (HTA) body, utility scores may need to be mapped from one instrument to another. According to a systematic review, only 63 of 303 studies in AD assessed QoL with 21 studies using the Dermatology Life Quality Index (DLQI), while only one study used the EQ-5D [7]. The DLQI was the first QoL assessment method developed specifically for dermatologic conditions, and it is the most widely used measure of patient-reported QoL outcomes in adults with AD in clinical trials [7–9]. Disease-specific measures such as the DLQI are sensitive to capturing disabilities caused by the disease and the impact of therapies on the disease [10], whereas generic preference-based measures such as the EQ-5D may not optimally capture important changes in disease-specific health attributes [6]. However, generic preference-based measures can be more sensitive than disease-specific measures when comparing the impact of comorbidities and side effects on utility values [11, 12]. Since the DLQI is not a preference-based measure, it cannot currently be used in cost–utility analysis (CUA) to capture HRQoL impacts when comparing interventions, which limits its applicability in health care resource allocation and ultimately treatment decision-making [6, 13–15]. Unlike the DLQI, the EQ-5D can be used to measure HRQoL across different diseases and interventions, and this standardization facilitates its use in economic evaluations [16]. When EQ-5D utility scores are absent from clinical studies, utility scores may be generated from other HRQoL measures through mapping [14]. Mapping consists of algorithm development, typically using regression modeling, to convert scores from one scale to another, such as DLQI to EQ-5D utilities [6, 17]. The first EQ-5D questionnaire that was developed consisted of three response levels indicating how much a disease affects certain aspects of a patient’s life [18]. This measure is widely used and have demonstrated reliability and validity [18]. A newer version with five response levels, the EQ-5D-5L, has been developed more recently [18], and crosswalk algorithms have been developed to convert between EQ-5D-5L and EQ-5D-3L [17]. In AD, as there is currently no available mapping algorithm to generate EQ-5D utility scores from DLQI scores, such an algorithm would be valuable for CUA of treatments for AD [15]. This paper reports the results of a direct mapping study that developed algorithms to map non-preference-based DLQI scores to preference-based EQ-5D-5L utility scores in patients with AD.

## Methods

Guidance from the Mapping onto Preference-based measures reporting Standards (MAPS) working group, the National Institute for Health and Care Excellence (NICE) Decision Support Unit, and the International Society for Pharmacoeconomics and Outcomes Research (ISPOR) were followed to ensure robust methodology [15, 19, 20].

### Study design and estimation sample

The mapping study described here was based on patients with AD from the United States (US), United Kingdom, France, and Germany, who participated in the National Health and Wellness Survey (NHWS) in 2015 and 2016. The NHWS includes condition-specific questions and patient-reported outcomes [21, 22]. Sex, age, and race (in the US) were incorporated into the NHWS sampling plan to ensure the sample was representative of the respective countries’ general populations. Participants for the NHWS were recruited through internal and external affiliate networks, eNewsletter campaigns, banners, co-registration with MySurvey.com partners, and through opt-in email. A total of 9579 NHWS participants were contacted and participants who did not provide written informed consent to participate in this study and were not able to complete the survey online were excluded. Participants who were not excluded were screened and selected based on the following inclusion criteria: diagnosed with AD and treated for it; currently suffer from mild, moderate, or severe AD according to the Patient-Oriented SCORAD which was included in the survey; self-reported age of ≥ 18 years or older on the NHWS; and must be from one of the four countries stipulated above. The estimation sample were chosen to fulfill quotas by disease severity and country, based on power calculations in order to include the population of interest and mimic the general AD population. According to the power calculations 130 or 173 respondents would be needed in each severity group to provide 80% or 90% power, respectively, to detect significant differences between disease severity levels. Inclusion criteria were not met by 7476 participants, 490 did not complete the screening, 357 had mild AD and were excluded because the mild quota was filled, and an additional 24 participants did not complete the survey [23]. Of the 1232 respondents that fulfilled the inclusion criteria, 134 had mild AD, 1098 had moderate-to-severe AD, and the sample size was sufficient to power this study [24]. For the present study, the estimation sample only included participants that completed both the DLQI and EQ-5D-5L questionnaires. The survey for the present study consisted of questions from validated questionnaires about AD. Questions about demographics, general health, and characteristics specific to AD were also included. The survey took approximately 35 min to complete and required answers to all questions to prevent missing data. The study was approved by the Institutional Review Board and Ethics Committees, and performed in accordance with various ethical principles [25], guidelines [26], practices [27], legislation, and regulations.

### Defining outcomes: source and target measures

For the mapping algorithm, the DLQI was the source measure from which utility scores for EQ-5D-5L, the target measure, were generated. The DLQI is a 10-item questionnaire that measures the impact of dermatology conditions and their treatments on HRQoL over the last one-week period [10]. DLQI items are scored from 0 to 3 (0 = “not at all” or “not relevant”; 1 = “a little”; 2 = “a lot”; 3 = “very much”) across aspects/domains (symptoms, feelings, daily and leisure activities, work or school, personal relationships, and treatment) to determine how much the condition and treatment has impacted the patient’s life [10]. The sum of the scores for the 10 items produces a DLQI score between 0 and 30, with higher scores indicating greater impairment in HRQoL [10]. The EQ-5D has been used to quantify HRQoL across a wide range of treatments and conditions and can be used to calculate quality-adjusted life years (QALY) for economic assessments [16, 28]. The EQ-5D consists of five dimensions (1 = Mobility; 2 = Self-care; 3 = Usual activities; 4 = Pain/Discomfort; and 5 = Anxiety/Depression), and two versions (EQ-5D-3L and EQ-5D-5L) [16]. Each dimension of the EQ-5D-5L consists of 5 levels (1 = “No problems”; 2 = “Slight problems”; 3 = “Moderate problems”; 4 = “Severe problems”; 5 = “Unable to”), with health states reported by the patient for the day the questionnaire is administered [28, 29].

The EQ-5D utility score is calculated by applying a preference-based population-specific value set with scores ranging from values less than 0 to 1 (negative values = health states considered worse than dead, with the specific lower bound dependent on country-specific mapping algorithm used; 0 = dead; and 1 = perfect health) [28–30]. The EQ-5D-5L was developed to address ceiling effects and improve sensitivity [31]. EQ-5D-5L data were collected from participants prior to NICE's updated recommendation in October 2019 to not use the 5L valuation set due to quality assurance concerns [5]. However, a key objective of this work was to map DLQI to EQ-5D utilities from a UK perspective, and because there is still only one UK 5L value set available, the value set for England specific to the EQ-5D-5L [30] was applied in this study. Based on the limitation of this value set, and because EQ-5D-3L scores were not available, EQ-5D-3L utility scores were calculated from the 5L utility scores by using a well-established crosswalk calculation proposed by Van Hout et al. [32] These results are presented in Online Appendix E. Additionally, an interactive companion tool has been developed (Online Resource 1) which will facilitate updates to the mapping once a new UK value set becomes available.

### Statistical analysis: exploratory data analysis—conceptual overlap

The degree to which there is a conceptual overlap between the DLQI and EQ-5D measures determines the rigor of the mapping algorithm [15]. Conceptual overlap is characterized by content similarity between the HRQoL outcome measures, if the same elements are not captured by the two measures, conceptual overlap will be lacking, and mapping will not be able to establish a relationship between DLQI and EQ-5D [13, 17]. Before investigating conceptual overlap, the total DLQI scores and EQ-5D utility scores were plotted for inspection, and tested for normality, skewness, and kurtosis. Because EQ-5D utility scores were not normally distributed, Spearman’s rank correlation coefficients were calculated between the total scores and respective domains of DLQI, and EQ-5D-5L to determine the degree of conceptual overlap (Table 1). Correlation strengths (very weak = 0–0.19; weak = 0.20–0.39; moderate = 0.40–0.59; strong = 0.60–0.79; and very strong = 0.80–1.00) were defined before analysis took place and used to interpret the Spearman’s rank correlation coefficient results [33].

**Table 1:** Spearman’s rank correlation coefficient matrix between DLQI and EQ-5D-5L

DLQI	Utility score					
	EQ-5D-5L	Mobility	Self-care	Usual activities	Pain/discomfort	Anxiety/depression
Total score	− 0.514	0.362	0.405	0.444	0.414	0.392
symptoms	− 0.337	0.255	0.237	0.311	0.326	0.213
Feelings	− 0.343	0.193	0.245	0.255	0.246	0.336
daily activities	− 0.364	0.311	0.337	0.343	− 0.266	0.248
Clothing	− 0.295	0.214	0.248	0.269	− 0.241	0.212
Social activities	− 0.441	0.339	0.346	0.382	0.325	0.330
Sport	− 0.310	0.241	0.258	0.328	0.272	0.171
Work and school	− 0.382	0.320	0.310	0.380	0.320	0.223
Personal relationship	− 0.323	0.255	0.292	0.310	0.225	0.226
Sex	− 0.290	0.251	0.229	0.278	0.241	0.162
Treatment	− 0.331	0.260	0.299	0.311	0.277	0.249

*DLQI* Dermatology Life Quality Index, *EQ-5D-5L*, EQ-5D-5-level

### Modeling approaches and performance

A variety of model types and independent variables/specifications were investigated with the aim of developing a simple, yet predictive model. Six model types (ordinary least squares [OLS], Tobit, three different two-part models, and a regression mixture model) and four different model specifications were considered (Table 2) to determine which model could best predict, and directly map EQ-5D-5L utility scores from the DLQI. OLS has some limitations such as predicting values beyond the EQ-5D feasible range, and under- or overestimation of utility scores at very good or very poor health, respectively [17], but given its simplicity and interpretability is frequently used in mapping [34]. Tobit models can censor predictions and were chosen because of the ceiling effect tendencies of centering values around 1 when modeling EQ-5D-5L utilities [17, 35]. To address the proportion of utilities at full health, two-part models were investigated in addition to traditional one-part models. Within the two-part models, a common first-stage logistic regression was used to estimate the probability of full health (disutility = 0), while a variety of forms were considered to model disutilities in the subset of individuals not at full health. Disutilities were then transformed back to utilities for comparison across all the mapping models. Some studies have found that mixture models outperformed OLS and Tobit models, in part because they address the multimodal distribution of EQ-5D values and abnormal distribution of values below 1 [17, 36]. Therefore, regression mixture modeling was performed as well. Regression mixture models aim to identify subgroups or clusters within data [37]. Heterogeneity of the relationships between predictors and outcomes can be modeled through identification of clusters within a distribution, and mixture models provide a semi-parametric and flexible approach to model “unknown distributional shapes” [36, 38]. The number of clusters the mixture model will employ needs to be pre-specified [38]. The number of clusters was increased from one until models stopped converging, and the Akaike Information Criteria (AIC) and Bayesian Information Criteria (BIC) of the models were used to determine the optimal number of clusters based on model fit (Online Appendix A). Models were fit assuming Gaussian mixture models. Parameter estimation was performed using the expectation maximization (EM) algorithm [39]. A set of regression parameters and accompanying standard errors were estimated for each cluster. Each of the six models were tested at four specification levels (level 1 used only the total DLQI score; level 2 used the total DLQI score and age and sex; level 3 and 4 were similar to levels 1 and 2 but used each of the DLQI items as categorical independent variables instead of the total DLQI score). Independent variables were limited in order to enable the algorithm to be applied to as many studies as possible, with as large a scope of datasets as possible.

**Table 2:** Summary of model types and levels

Type of model	Prediction	Modeling distribution
OLS	Utility	Normal
Tobit	Utility	Normal
Two-part: GLM (1)–OLS (2)	Disutility	Part 1: binomial, part 2: normal
Two-part: GLM (1)–OLS (2)	Disutility	Part 1: binomial, part 2: lognormal
Two-part: GLM (1)–GLM (2)	Disutility	Part 1: binomial, part 2: gamma
Regression mixture	Utility	Normal

Level	Available data	Independent variables
Level 1	Total DLQI	Total DLQI
Level 2	Total DLQI, age, and sex	Total DLQI, age, and sex
Level 3	DLQI items	DLQI1, DLQI 2, DLQI3, DLQI4, DLQI5, DLQI6, DLQI7, DLQI8, DLQI9, DLQI10
Level 4	DLQI items, age, and sex	DLQI1, DLQI 2, DLQI3, DLQI4, DLQI5, DLQI6, DLQI7, DLQI8, DLQI9, DLQI10, age, sex

*DLQI* Dermatology Life Quality Index, *GLM* Generalized Linear Model, *OLS* Ordinary Least Squares, *DLQI 1* symptoms, *DLQI 2* feelings, *DLQI 3* daily activities, *DLQI 4* clothing, *DLQI 5* social activities, *DLQI 6* sport, *DLQI 7* work and school, *DLQI 8* personal relationship, *DLQI 9* sex, *DLQI 10* treatment

Model diagnostic criteria were used to evaluate model performance, specifically how well observed utility scores can be predicted by the mapping models, and included root mean squared error (RMSE) and mean absolute error (MAE) [40]. MAE is a linear score that assigns an equal weight to all observations, and it is an indication of the average size of the error rather than its direction; and the RMSE is more sensitive than the MAE to large errors/outliers, and better able to assess differences between model performances, with higher scores indicating worse model performance [15, 17, 40]. In addition to these summary measures (RMSE, MAE), model fit to select the optimal number of clusters for the regression mixture model was described by using AIC and BIC [36]. The overall ranking was based on first ranking the models independently by RMSE and MAE, and then taking the average ranking of each model between these two criteria. R3.3.3 (R Core Team, Vienna, Austria) was used to estimate all regression models and additional statistical tests [41], apart from the mixture model for which the *flexmix* (V2.3–15) package in *R* (V3.6.1) was used [42].

## Results

### Descriptive statistics: characterizing the cohort

**Fig. 1:**
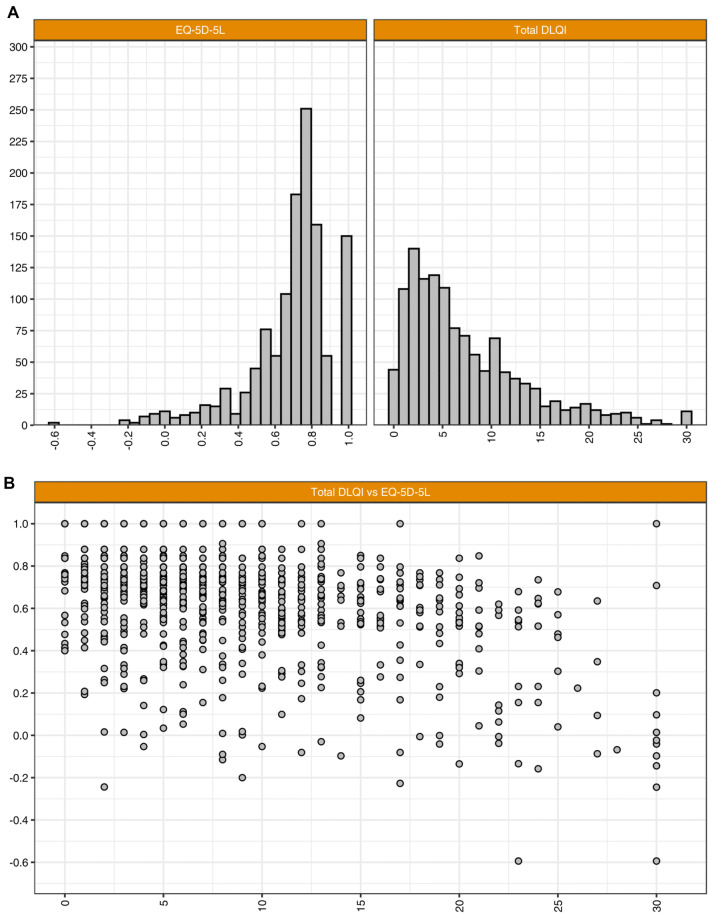
EQ-5D-5L and DLQI scores and the relationship between these measures. **a** Probability distributions for the EQ-5D-5-level (EQ-5D-5L) utility scores and the Dermatology Life Quality Index (DLQI) scores. **b** Scatter plot of the EQ-5D-5L utility scores and DLQI scores where size of points corresponds to the number of observations

Descriptive statistics are presented in Table 3, and EQ-5D-5L and DLQI scores and the relationship between these measures are presented in Fig. 1. The final estimation sample consisted of 1,232 participants with AD that completed the web-based survey, WITH a mean age of 48.3 years, and most participants were female (67%). EQ-5D-5L had a mean score of 0.78 and a median score of 0.83. The mean DLQI score was 7.23 and the median score was 5.

**Table 3:** Summary of descriptive statistics of the final sample

Descriptive statistics	
Characteristics	Estimation sample (out of 9759)
Respondents (*n*)	1232
Age (mean ± SD)	48.28 ± 14.98
Women (proportion)	67.37%
EQ-5D-5L (mean ± SD)	0.78 ± 021
Median	0.83
Kurtosis	3.55
Skewness	− 1.73
Shapiro Wilk	*p* value < 2.2e^−16^
DLQI score (mean ± SD)	7.23 ± 6.19
Median	5
Kurtosis	1.37
Skewness	4.63
Shapiro Wilk	*p* value < 2.2e^−16^

*DLQI* Dermatology Life Quality Index, *EQ-5D-5L* EQ-5D-5-level, *SD* standard deviation

### Conceptual overlap

Spearman’s rank correlation coefficients between the total DLQI score and EQ-5D-5L utility scores and domains are reported in Table 1. Conceptually, EQ-5D and DLQI are negatively correlated (lower DLQI scores and higher EQ-5D utility scores indicate better health), and moderately negative correlations (EQ-5D-5L *p* = − 0.514) between EQ-5D utility scores and DLQI total scores were observed. There was a moderately negative correlation between EQ-5D utility scores and the “Social Activities” DLQI domain (EQ-5D-5L = − 0.441), and weak negative correlations between EQ-5D scores and the remaining DLQI items (*p* scores between − 0.20 and − 0.39). The highest (moderately) correlated EQ-5D domains with total DLQI scores were “Usual Activities” (0.444), “Pain/Discomfort” (0.414), and “Self-care” (0.405), while “Mobility” (0.362) and “Anxiety/Depression” (0.392) were weakly correlated with the total DLQI scores. There was a weaker correlation between DLQI items and EQ-5D utility scores compared to EQ-5D dimensions and total DLQI score.

### Model selection and performance

**Fig. 2:**
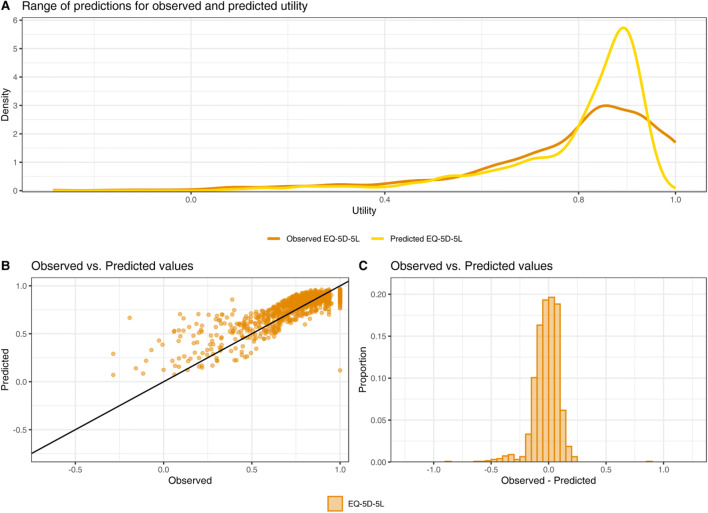
Graphical representation of model performance depicting EQ-5D-5L observed versus predicted values. **a** Range of predictions for observed and predicted EQ-5D-5-level (EQ-5D-5L) utilities. **b** Scatter plot of the range of predictions for observed versus predicted EQ-5D-5L utility values. **c** Histogram representing error distribution of the observed versus predicted EQ-5D-5L utility values

Model performance is based on the mapping model predictions compared to the actual EQ-5D-5L utility scores. Coefficients for generating EQ-5D-5L utility scores from DLQI scores for the best model by level/specification are presented in Online Appendix B, including standard errors. Additionally, an app is provided in Online Resource 1 to assist with converting DLQI scores from de novo samples to EQ-5D -5L utility scores. Regression mixture models were on average the best models for predicting EQ-5D-5L utility scores from DLQI scores (Table 4). Model two, total DLQI with age and sex as independent variables was the best overall with the second lowest RMSE (0.113), and lowest MAE (0.079) for EQ-5D-5L. The regression mixture model with total DLQI, and age and sex as independent variables predicted EQ-5D-5L values ranging from 0.071 to 0.969. There was a drastic increase in both AIC and BIC when moving from one to two clusters for all models, but fit worsened overall and inconsistency between models increased when moving to more than two clusters. Therefore, it was determined that two clusters were optimal. Cluster sizes tended to be consistent between all models, with approximately 80% of the observations falling in cluster 1 and 20% in cluster 2. The larger cluster contained the larger EQ-5D values and tended to fit the data very well, while the smaller cluster was widely spread out among the smaller observations and fit the data quite poorly. Due to the multimodal nature of the distribution of the EQ-5D values, allowing separate sets of parameters to be fit to different parts of the data vastly improved the model fit compared to forcing all the data to be described by a single set of parameters. The proportion of predictions within ± 0.05, ± 0.10, and ± 0.15 of the observed EQ-5D-5L utility scores is reported in Online Appendix C to provide more information about the predictive ability of the models. Errors were more prominent in the lower range of the data (− 0.25, 0.5) compared to the higher values (0.5, 1). Figure 2 demonstrates the predictive ability of the regression mixture model with total DLQI with age and sex as independent variables, where 38.96% of the EQ-5D-5L predictions were within ± 0.05 of the observed values, and 74.11% EQ-5D-5L were within ± 0.10. Figure 2 is a graphical representation of model performance—predicted versus observed EQ-5D utility scores—with element B of the figure showing the range in predicted vs. observed values, and element C showing error distribution. The two-part models were the only models that predicted values below 0 and values of 1 (perfect health). The regression mixture models at all levels had the most predictions within ± 0.05, ± 0.10, and ± 0.15, and were higher ranked at each level than the OLS, Tobit, and Two-part models.

**Table 4:** Model performance for each models and level

Regression type	Mean	SD	EQ-5D-5L				
			Min	Max	RMSE	MAE	Rank
Observed EQ-5D-5L	0.777	0.2097	− 0.285	1			
Total DLQI							
OLS	0.776	0.112	0.366	0.907	0.178	0.127	12
Tobit	0.791	0.122	0.342	0.933	0.178	0.124	11
Two-part: GLM (logistic)–OLS (normal)	0.793	0.202	− 0.029	1.424	0.266	0.198	20
Two-part: GLM (logistic)–OLS (lognormal)	0.816	0.128	0.304	1	0.230	0.158	13
Two-part: GLM (logistic)–GLM (gamma)	0.825	0.142	− 0.167	1	0.253	0.180	17
Regression mixture	0.794	0.158	0.108	0.933	0.113	0.080	2
Total DLQI + age + sex							
OLS	0.776	0.116	0.342	0.972	0.175	0.125	10
Tobit	0.791	0.127	0.313	1.010	0.176	0.123	9
Two-part: GLM (logistic)–OLS (normal)	0.793	0.202	0.046	1.313	0.266	0.198	20
Two-part: GLM (logistic)–OLS (lognormal)	0.816	0.128	0.244	1	0.230	0.158	13
Two-part: GLM (logistic)–GLM (gamma)	0.818	0.150	− 0.071	1	0.257	0.186	18
Regression Mixture	0.794	0.161	0.071	0.969	0.113	0.079	1
DLQI items							
OLS	0.776	0.130	0.233	1.012	0.165	0.118	8
Tobit	0.790	0.141	0.228	1.079	0.166	0.117	5
Two-part: GLM (logistic)–OLS (normal)	0.793	0.202	− 0.063	1.335	0.266	0.198	20
Two-part: GLM (logistic)–OLS (lognormal)	0.816	0.128	0.210	1	0.230	0.158	13
Two-part: GLM (logistic)–GLM (gamma)	0.818	0.160	− 0.792	1	0.260	0.181	18
Regression mixture	0.790	0.163	0.140	0.994	0.113	0.083	4
DLQI items + age + sex							
OLS	0.776	0.131	0.220	1.003	0.164	0.118	5
Tobit	0.791	0.142	0.218	1.070	0.166	0.117	5
Two-part: GLM (logistic)–OLS (normal)	0.793	0.202	0.007	1.319	0.266	0.198	20
Two-part: GLM (logistic)–OLS (lognormal)	0.816	0.128	0.182	1	0.230	0.158	13
Two-part: GLM (logistic)–GLM (gamma)	0.820	0.168	− 0.809	1	0.267	0.187	24
Regression mixture	0.791	0.163	0.085	0.996	0.112	0.082	2

*DLQI* Dermatology Life Quality Index, *EQ-5D-5L* EQ-5D-5-level, *GLM* Generalized Linear Model, *MAE* mean absolute error, *ME* mean error, *OLS* Ordinary Least Squares, *RMSE* root mean squared error, *SD* standard deviation

### Uncertainty

Variance–covariance matrices were calculated and reported in Online Appendix D for all independent variables from the highest ranked model for each level to account for the uncertainty associated with direct mapping, and allow for probabilistic sensitivity analysis in a CUA [20]. The standard error of coefficients can be calculated from the variance–covariance matrix and used in combination with the coefficients, and model types and specifications to characterize the distribution of model inputs required for probabilistic sensitivity analysis [20].

### Face validity

Most coefficients had expected signs, reflecting a relationship in which worse health as measured by the DLQI was associated with lower utility scores for EQ-5D-5L [15]. When the overall DLQI score was considered in the level 1 and 2 models, this expected relationship held consistently. In the level 3 and 4 models for which the DLQI items were used as independent variables, there were several examples of a worse score indicating better health for individual items. However, in all such cases, coefficients were relatively small in magnitude and none were statistically significant, indicating that the models were likely describing a lack of association rather than a true implausible relationship. Additionally, there was one case of inconsistency, where a patient indicated debilitating skin problems on the DLQI (DLQI = 30) but indicated perfect health on the EQ-5D-5L (EQ-5D-5L = 1).

## Discussion

In this study, mapping algorithms were developed to generate EQ-5D-5L utility scores from DLQI total scores or item scores (with/without age and sex as independent variables) in patients with AD. RMSE and MAE were used to rank 24 models (4 levels of input for 6 model types) according to their ability to predict EQ-5D-5L utility scores from DLQI data. The best performing model with the highest average accuracy for predicting EQ-5D-5L utility scores was a regression mixture model with total DLQI plus age and sex as independent variables.

This study primarily included patients with moderate-to-severe AD and the algorithm developed in this study is therefore only applicable to similar populations. To the knowledge of the authors, this is the first study to map from DLQI to EQ-5D-5L exclusively in patients with AD. While previous studies have developed mappings from DLQI to EQ-5D-3L, they have been restricted to linear regression methods in populations with psoriasis [43–46]. Davison et al. investigated different mapping models in patients with psoriasis, and found that an OLS model with DLQI items, age, and sex was the best performing model to map from DLQI to EQ-5D-3L [13]. Ali et al. used an ordinal logistic regression (OLR) model, based on MAE and mean square error (MSE), and utilized response mapping from DLQI to EQ-5D-3L in patients with various dermatologic conditions (6.7% of patients had AD) [47]. The ranges of the EQ-5D-3L and EQ-5D-5L are different, which causes the ME, MAE, and RMSE to vary as they are absolute measures that depend on the total range of values. Therefore, it is not feasible to compare the EQ-5D-5L models in this study with the EQ-5D-3L models in the study by Ali et al. Ali et al. applied the UK tariff value set [47], and there were differences in population that should be considered. The study sample in Ali et al. was more heterogeneous (multiple skin conditions and participants from 13 countries) [47] which might have resulted in large in-sample variations, making direct comparisons challenging.

This study provides new information about the relationship between DLQI and EQ-5D-5L utility scores, and the coefficients necessary to calculate these utility scores, allowing studies that have only collected DLQI data to be included in CUA. The models that were developed included different model levels, which enabled the use of different information sources (total DLQI, DLQI items, age, and sex) to predict EQ-5D utility scores. Information sources were selected based on relevance and availability (from publications and clinical studies) so the algorithms could be applied more widely.

Limitations included inconsistencies in answers between the DLQI and EQ-5D-5L questionnaires. One participant reported debilitating skin problems on the DLQI but perfect health on the EQ-5D-5L, and it is unlikely that their AD did not affect their general HRQoL. To avoid selection bias, these data were included in the analysis. The opposite was also true for some participants, and this could be explained by comorbidities. Participants with comorbidities were not excluded, and many participants had AD-related and non-AD-related comorbidities. Comorbid conditions can decrease HRQoL on the EQ-5D-5L [48], but is unlikely to affect the DLQI, therefore, it may weaken the correlation between EQ-5D and DLQI. However, excluding participants with comorbidities may make the study sample less representative of the general AD population, which is likely to have comorbidities [49, 50]. Additionally, because different HRQoL aspects are measured by the EQ-5D and DLQI, they are complementary, with the DLQI more sensitive to changes in clinical outcomes related to AD that can affect HRQoL, and the EQ-5D being more broad to allow comparisons of HRQoL between different diseases and taking the effects of comorbidities into consideration [48]. Misinterpretation or misunderstandings while responding to surveys is a fundamental limitation [51]. The best performing models were able to accurately predict the mean EQ-5D-5L. One limitation of mixture models is that there is a risk that clusters can be over extracted if outlier or non-normality is present [36]. However, the number of clusters in this study was pre-specified at two, and the response variable was almost completely contained between 0 and 1. Therefore, there was little chance that outliers were present in this space. Another limitation surrounding mixture models is the additional complexity needed to estimate multiple sets of parameters for each cluster [42]. Finally, predictions using the mixture models are most appropriate when used at a population or subgroup level and have limited utility when used to predict at the individual level [37, 38, 52].

Future extensions of this work could include mapping algorithms for additional country-specific EQ-5D utility value sets, to develop algorithms applicable to other populations. In addition, when suitable datasets become available mapping between DLQI and EQ-5D-5L in patients with different levels of AD severity will be investigated, as well as external validation of the results described here based on an alternative data source (NCT03725722) [53] that includes both DLQI and EQ-5D for individuals with AD. Other models (i.e., non-regression) could also be explored in mapping from DLQI to EQ-5D [54].

## Conclusions

It is preferable to use utilities derived from directly administering the EQ-5D-3L or EQ-5D-5L, but mapping algorithms are useful when these data are not available. This study developed mapping algorithms that can be used to predict EQ-5D-5L utility scores from DLQI scores with reasonable accuracy in patients with moderate-to-severe AD. The mapping model with the best predictive ability was the regression mixture model with total DLQI plus age and sex as independent variables. This was the first study of its kind exclusively in patients with AD, and this mapping algorithm can be used in economic evaluations to calculate EQ-5D-5L utility scores when they are unavailable in order to determine the impact of treatments for AD on QALY and assist HTAbodies by determining the economic value of treatments.

## Electronic supplementary material

Below is the link to the electronic supplementary material.

Supplementary file1 (DOCX 86 kb)
