# Supplementary material for: Mapping Dermatology Life Quality Index (DLQI) scores to EQ-5D utility scores using data of patients with atopic dermatitis from the National Health and Wellness Study
Source: Qual Life Res. 2020 Apr 15;29(9):2529–39. doi: 10.1007/s11136-020-02499-1 (PMC7434755; doi:10.1007/s11136-020-02499-1)
Supplement: Supplementary file 1 — Supplementary file1 (DOCX 86 kb) [file 11136_2020_2499_MOESM1_ESM.docx]

**Supplemental Materials:** Online resource 1 can be found at https://dlqi.broadstreetheor.com/

**Appendix A Table A1. Akaike Information Criteria and Bayesian**

**Information Criteria values by Model and Cluster**

| **Outcome** | | **EQ-5D-5L** | |
| --- | --- | --- | --- |
| **Model** | **Clusters** | **AIC** | **BIC** |
| Model 1 | 1 | -757.32 | -741.98 |
| Model 1 | 2 | -1314.37 | -1278.56 |
| Model 1 | 3 | -1365.77 | -1309.49 |
| Model 1 | 4 | -1394.02 | -1317.27 |
| Model 1 | 5 | -1403.77 | -1306.56 |
| Model 1 | 6 | -1411.65 | -1293.97 |
| Model 2 | 1 | -791.08 | -765.50 |
| Model 2 | 2 | -1353.05 | -1296.77 |
| Model 2 | 3 | -1396.95 | -1309.97 |
| Model 2 | 4 | -1433.80 | -1316.12 |
| Model 2 | 5 | -1462.31 | -1313.94 |
| Model 2 | 6 | -1436.48 | -1288.10 |
| Model 3 | 1 | -680.05 | -618.65 |
| Model 3 | 2 | -1216.44 | -1088.53 |
| Model 3 | 3 | -1294.70 | -1100.28 |
| Model 3 | 4 | -1333.58 | -1072.64 |
| Model 3 | 5 | -1375.67 | -1048.22 |
| Model 3 | 6 | -1364.42 | -970.45 |
| Model 4 | 1 | -689.89 | -618.26 |
| Model 4 | 2 | -1230.54 | -1082.17 |
| Model 4 | 3 | -1308.42 | -1083.30 |
| Model 4 | 4 | -1321.02 | -1019.15 |
| Model 4 | 5 | -1330.41 | -1028.54 |
| Model 4 | 6 | -1370.55 | -915.20 |

AIC, Akaike Information Criteria; BIC, Bayesian Information Criteria

**Appendix B Table B1. Model coefficients and standard errors**

| **EQ-5D-5L Model Coefficients - Regression Mixture Models** | | | | | | |
| --- | --- | --- | --- | --- | --- | --- |
|  | | **Cluster 1** | | **Cluster 2** | | |
| **Level 1 - Total DLQI** | | | | | | |
| **Cluster Size** | | **n** | **%** | **n** | **%** | |
|  |  | 988 | 80.2 | 244 | 19.8 | |
| **Coefficients** | | **Estimate** | **SE** | **Estimate** | **SE** | |
| **Intercept** | | 0.933 | 0.005 | 0.744 | 0.027 | |
| **Total DLQI** | | -0.011 | 0.001 | -0.021 | 0.002 | |
| **Level 2 - Total DLQI, age and sex** | | | | | | |
| **Cluster Size** | | **n** | **%** | **n** | **%** | |
|  |  | 988 | 80.2 | 244 | 19.8 | |
| **Coefficients** | | **Estimate** | **SE** | **Estimate** | **SE** | |
| **Intercept** | | 0.976 | 0.013 | 0.943 | 0.064 | |
| **Total DLQI** | | -0.012 | 0.001 | -0.023 | 0.002 | |
| **Age** | | -0.001 | 0.000 | -0.004 | 0.001 | |
| **Sex** | | 0.014 | 0.007 | -0.007 | 0.030 | |
| **Level 3 - DLQI Items** | | | | | | |
| **Cluster Size** | | **n** | **%** | **n** | **%** | |
|  |  | 959 | 77.8 | 273 | 22.2 | |
| **Coefficients** | | **Estimate** | **SE** | **Estimate** | **SE** | |
| **Intercept** | | 1.106 | 0.046 | 1.019 | 0.013 | |
| **DLQI Score** | | | |  |  | |
| **DLQI Items** |  | **Estimate** | **SE** | **Estimate** | **SE** | |
|  | **DLQI 1** | -0.050 | -0.020 | -0.026 | 0.005 | |
|  | **DLQI 2** | -0.045 | 0.017 | -0.029 | 0.005 | |
|  | **DLQI 3** | -0.012 | 0.008 | 0.001 | 0.002 | |
|  | **DLQI 4** | -0.007 | 0.009 | 0.006 | 0.002 | |
|  | **DLQI 5** | -0.026 | 0.010 | -0.008 | 0.002 | |
|  | **DLQI 6** | 0.000 | 0.005 | 0.000 | 0.001 | |
|  | **DLQI 7** | -0.053 | 0.010 | -0.016 | 0.002 | |
|  | **DLQI 8** | 0.000 | 0.007 | 0.000 | 0.002 | |
|  | **DLQI 9** | -0.011 | 0.005 | -0.001 | 0.001 | |
|  | **DLQI 10** | -0.014 | 0.011 | 0.001 | 0.002 | |
| **Level 4 - DLQI Items, age and sex** | | | | | | |
| **Cluster Size** | | **n** | **%** | **n** | **%** | |
|  |  | 970 | 78.7 | 262 | 21.3 | |
| **Coefficients** | | **Estimate** | **SE** | **Estimate** | **SE** | |
| **Intercept** | | 1.053 | 0.019 | 1.162 | 0.072 | |
| **Age** | | -0.001 | 0.000 | -0.001 | 0.001 | |
| **Sex** | | 0.016 | 0.007 | 0.003 | 0.027 | |
| **DLQI Score** | | | | | | |
| **DLQI Items** |  | **Estimate** | **SE** | **Estimate** | **SE** |  |
|  | **DLQI 1** | -0.025 | 0.005 | -0.048 | 0.020 |  |
|  | **DLQI 2** | -0.034 | 0.005 | -0.046 | 0.018 |  |
|  | **DLQI 3** | 0.000 | 0.002 | -0.014 | 0.008 |  |
|  | **DLQI 4** | 0.007 | 0.002 | -0.007 | 0.009 |  |
|  | **DLQI 5** | -0.008 | 0.002 | -0.026 | 0.010 |  |
|  | **DLQI 6** | 0.001 | 0.001 | 0.001 | 0.005 |  |
|  | **DLQI 7** | -0.015 | 0.002 | -0.051 | 0.011 |  |
|  | **DLQI 8** | 0.000 | 0.002 | -0.001 | 0.007 |  |
|  | **DLQI 9** | - 0.001 | 0.001 | -0.011 | 0.005 |  |
|  | **DLQI 10** | -0.001 | 0.002 | -0.018 | 0.013 |  |

DLQI, Dermatology Life Quality Index; EQ-5D-5L, EQ-5D-5-level, SE, standard error

Note: p values for the mixture model are not provided because they should not be used in the traditional sense, and only in an exploratory manner.[42] P values will be inaccurate because they have not been adjusted for the fact that the same data are used to select the model and compute the p-values.[42]

**Appendix C Table C1. Summary of the proportion of mapping estimates for each of the models**

| **EQ-5D-5L** | | | |
| --- | --- | --- | --- |
| **Regression type by Data Level** | **±0.05** | **±0.10** | **±0.15** |
| Data Level 1 Total DLQI | | | |
| OLS | 29.30% | 52.19% | 82.06% |
| Tobit | 31.49% | 56.66% | 81.66% |
| Two-part: GLM (Logistic) - OLS (Normal) | 16.15% | 32.87% | 61.69% |
| Two-part: GLM (Logistic) - OLS (Lognormal) | 25.32% | 47.81% | 78.25% |
| Two-part: GLM (Logistic) - GLM (Gamma) | 21.02% | 39.69% | 69.56% |
| Regression Mixture | 40.83% | 72.65% | 88.80% |
| Data Level 2 Total DLQI Age and Sex | | | |
| OLS | 28.73% | 53.57% | 82.55% |
| Tobit | 30.44% | 56.25% | 82.39% |
| Two-part: GLM (Logistic) - OLS (Normal) | 16.40% | 32.31% | 60.88% |
| Two-part: GLM (Logistic) - OLS (Lognormal) | 24.51% | 48.30% | 77.44% |
| Two-part: GLM (Logistic) - GLM (Gamma) | 20.05% | 38.64% | 66.23% |
| Regression Mixture | 38.96% | 74.11% | 90.34% |
| Data Level 3 DLQI Items | | | |
| OLS | 31.01% | 54.95% | 83.77% |
| Tobit | 31.74% | 58.60% | 83.36% |
| Two-part: GLM (Logistic) - OLS (Normal) | 18.02% | 34.33% | 62.82% |
| Two-part: GLM (Logistic) - OLS (Lognormal) | 23.78% | 45.70% | 75.41% |
| Two-part: GLM (Logistic) - GLM (Gamma) | 21.83% | 42.13% | 68.75% |
| Regression Mixture | 38.64% | 70.29% | 87.99% |
| Data Level 4 DLQI Items Age and Sex | | | |
| OLS | 30.76% | 56.09% | 84.09% |
| Tobit | 31.82% | 56.98% | 83.52% |
| Two-part: GLM (Logistic) - OLS (Normal) | 17.78% | 34.01% | 61.04% |
| Two-part: GLM (Logistic) - OLS (Lognormal) | 24.11% | 46.59% | 75.73% |
| Two-part: GLM (Logistic) - GLM (Gamma) | 21.35% | 41.64% | 67.86% |
| Regression Mixture | 38.80% | 70.86% | 87.66% |

DLQI, Dermatology Life Quality Index; EQ-5D-5L, EQ-5D-5-level; GLM, Generalized Linear Model;

OLS, Ordinary Least Squares

**Appendix D**

**Table D1. Regression Mixture Model 1 – Total DLQI**

**Variance-Covariance Matrix**

| Coefficients | Intercept | Total DLQI | Intercept | Total DLQI |
| --- | --- | --- | --- | --- |
| Intercept | 2.45985E-05 | -2.06236E-06 | 1.09687E-05 | 4.19266E-07 |
| Total DLQI | -2.06236E-06 | 3.51294E-07 | 1.17341E-06 | -6.82817E-08 |
| Intercept | 1.09687E-05 | 1.17341E-06 | 0.000711468 | -3.38547E-05 |
| Total DLQI | 4.19266E-07 | -6.82817E-08 | -3.38547E-05 | 3.82683E-06 |

**Blue = Cluster 1; Green = Cluster 2**

DLQI, Dermatology Life Quality Index

**Table D2. Regression Mixture Model 2 – Total DLQI, age and sex**

**Variance-Covariance Matrix**

| Coefficients | Intercept | Total DLQI | Age | Gender | Intercept | Total DLQI | Age | Sex |
| --- | --- | --- | --- | --- | --- | --- | --- | --- |
| Intercept | 0.00016923 | -2.6881E-06 | -2.4894E-06 | -3.9451E-05 | -1.4139E-05 | 4.615E-07 | 2.9853E-07 | -3.379E-06 |
| Total DLQI | -2.6881E-06 | 3.381E-07 | 1.6053E-08 | -7.9699E-08 | 1.4613E-06 | -5.2555E-08 | 1.0819E-08 | -1.3924E-06 |
| Age | -2.4894E-06 | 1.6053E-08 | 4.8008E-08 | 2.4945E-07 | 3.495E-07 | 5.3069E-09 | -7.1293E-09 | 1.9398E-07 |
| Sex | -3.9451E-05 | -7.9699E-08 | 2.4945E-07 | 4.3151E-05 | -4.5188E-06 | -6.0121E-07 | 1.6684E-07 | 3.2391E-06 |
| Intercept | -1.4139E-05 | 1.4613E-06 | 3.495E-07 | -4.5188E-06 | 0.00408478 | -4.6607E-05 | -5.5969E-05 | -0.00088249 |
| Total DLQI | 4.615E-07 | -5.2555E-08 | 5.3069E-09 | -6.0121E-07 | -4.6607E-05 | 3.644E-06 | 2.2076E-07 | 4.8068E-06 |
| Age | 2.9853E-07 | 1.0819E-08 | -7.1293E-09 | 1.6684E-07 | -5.5969E-05 | 2.2076E-07 | 1.047E-06 | 4.4614E-06 |
| Sex | -3.379E-06 | -1.3924E-06 | 1.9398E-07 | 3.2391E-06 | -0.00088249 | 4.8068E-06 | 4.4614E-06 | 0.00092671 |

**Blue = Cluster 1; Green = Cluster 2**

DLQI, Dermatology Life Quality Index

**Table D3. Regression Mixture Model 3– DLQI items**

**Variance-Covariance Matrix**

| 4 | Intercept | DLQI1 | DLQI2 | DLQI3 | DLQI4 | DLQI5 | DLQI6 | DLQI7 | DLQI8 | DLQI9 | DLQI10 | Intercept | DLQI1 | DLQI2 | DLQI3 | DLQI4 | DLQI5 | DLQI6 | DLQI7 | DLQI8 | DLQI9 | DLQI10 |
| --- | --- | --- | --- | --- | --- | --- | --- | --- | --- | --- | --- | --- | --- | --- | --- | --- | --- | --- | --- | --- | --- | --- |
| Intercept | 0.002079515 | -0.00054627 | -0.00013248 | -2.6882E-05 | -5.3446E-05 | 1.39511E-05 | -4.4555E-05 | 7.0785E-05 | 1.31493E-05 | -3.66E-05 | 1.47951E-05 | -2.5951E-05 | -9.2918E-08 | 8.66492E-06 | 4.60335E-07 | 6.76283E-07 | 9.38987E-07 | -2.426E-07 | 1.40449E-06 | -3.9619E-07 | 1.00112E-06 | 4.5635E-07 |
| DLQI1 | -0.00054627 | 0.000407757 | -9.9297E-05 | -2.1839E-06 | -1.2923E-05 | -1.4815E-05 | 7.90477E-06 | -7.0943E-05 | 8.40463E-06 | -2.4434E-06 | -1.9711E-05 | 3.88095E-06 | -4.9634E-07 | -3.9794E-06 | -9.4326E-08 | 4.66334E-07 | -1.7369E-07 | 2.00807E-07 | 7.52623E-07 | 3.1448E-07 | -1.54E-07 | 1.58792E-08 |
| DLQI2 | -0.00013248 | -9.9297E-05 | 0.000303115 | -1.2998E-07 | -2.6292E-05 | -5.5511E-05 | 9.68782E-06 | 1.81623E-05 | -2.5342E-05 | 1.20947E-05 | -7.2231E-05 | 3.77096E-06 | -4.4113E-06 | 3.49661E-06 | -2.1892E-07 | 1.08788E-06 | -2.7575E-06 | -6.0385E-07 | 3.75028E-06 | -2.3487E-06 | 6.88862E-07 | -2.1259E-07 |
| DLQI3 | -2.6882E-05 | -2.1839E-06 | -1.2998E-07 | 6.77684E-05 | -9.3834E-07 | -1.6099E-05 | -6.2839E-06 | -9.6412E-06 | -9.0018E-06 | 6.76402E-07 | -7.2112E-06 | 1.64203E-07 | 3.15516E-07 | -4.5709E-07 | 1.11866E-06 | 1.5862E-07 | -6.3983E-07 | -7.592E-09 | -4.5795E-07 | -2.6406E-08 | 1.24211E-07 | -1.3841E-09 |
| DLQI4 | -5.3446E-05 | -1.2923E-05 | -2.6292E-05 | -9.3834E-07 | 7.61689E-05 | -4.0956E-06 | -1.2211E-06 | -1.1135E-05 | -1.2613E-06 | 2.71397E-06 | -3.413E-06 | 2.8328E-06 | 7.31719E-07 | -1.5184E-06 | -4.2958E-08 | 1.74595E-06 | -7.0481E-07 | 8.11958E-08 | -1.0072E-06 | 2.27867E-07 | -2.2064E-07 | -5.3768E-07 |
| DLQI5 | 1.39511E-05 | -1.4815E-05 | -5.5511E-05 | -1.6099E-05 | -4.0956E-06 | 0.000102281 | -4.6855E-06 | -1.358E-05 | 5.15816E-06 | -3.8549E-06 | 7.06391E-06 | 1.98327E-07 | 9.72248E-07 | -2.4386E-07 | -5.5029E-07 | -2.3445E-06 | 4.42427E-06 | 4.08429E-07 | -2.3304E-06 | 2.16821E-06 | -6.5075E-07 | -8.0133E-08 |
| DLQI6 | -4.4555E-05 | 7.90477E-06 | 9.68782E-06 | -6.2839E-06 | -1.2211E-06 | -4.6855E-06 | 2.07148E-05 | -1.4019E-05 | -3.8235E-06 | -1.8875E-06 | -2.5965E-06 | 1.05097E-06 | -3.5121E-08 | -4.6603E-07 | -4.74E-08 | 7.90209E-08 | -8.5942E-08 | -4.8603E-09 | -2.3889E-07 | 9.44947E-08 | -3.7288E-08 | -1.4514E-07 |
| DLQI7 | 7.0785E-05 | -7.0943E-05 | 1.81623E-05 | -9.6412E-06 | -1.1135E-05 | -1.358E-05 | -1.4019E-05 | 0.000108667 | -1.1629E-05 | -2.0264E-06 | -1.6242E-05 | -4.6211E-06 | 1.08293E-06 | 2.16082E-06 | -1.5482E-07 | 8.52675E-08 | -1.4495E-06 | -4.0038E-07 | 2.74108E-06 | -8.0007E-07 | 2.9714E-07 | -8.0335E-07 |
| DLQI8 | 1.31493E-05 | 8.40463E-06 | -2.5342E-05 | -9.0018E-06 | -1.2613E-06 | 5.15816E-06 | -3.8235E-06 | -1.1629E-05 | 4.80123E-05 | -1.3018E-05 | 5.04031E-06 | -1.8208E-06 | 9.83537E-07 | -6.1592E-07 | -1.4928E-07 | -7.345E-07 | 1.53691E-06 | 3.89446E-07 | -7.4539E-07 | 1.75134E-06 | -7.2339E-07 | -2.6193E-07 |
| DLQI9 | -3.66E-05 | -2.4434E-06 | 1.20947E-05 | 6.76402E-07 | 2.71397E-06 | -3.8549E-06 | -1.8875E-06 | -2.0264E-06 | -1.3018E-05 | 2.21165E-05 | -3.8205E-06 | 6.9435E-07 | -3.9044E-07 | 3.42196E-07 | 3.46663E-08 | 2.10116E-08 | -2.2866E-07 | -1.2279E-07 | 3.79813E-07 | -6.006E-07 | 5.87469E-07 | 3.69248E-07 |
| DLQI10 | 1.47951E-05 | -1.9711E-05 | -7.2231E-05 | -7.2112E-06 | -3.413E-06 | 7.06391E-06 | -2.5965E-06 | -1.6242E-05 | 5.04031E-06 | -3.8205E-06 | 0.000121993 | -3.6802E-06 | 2.17048E-07 | 2.01822E-06 | -5.6187E-09 | -1.3137E-06 | 1.16966E-06 | 9.54603E-08 | -1.9882E-06 | -4.5729E-08 | 1.20064E-07 | 3.36554E-06 |
| Intercept | -2.5951E-05 | 3.88095E-06 | 3.77096E-06 | 1.64203E-07 | 2.8328E-06 | 1.98327E-07 | 1.05097E-06 | -4.6211E-06 | -1.8208E-06 | 6.9435E-07 | -3.6802E-06 | 0.000165971 | -4.1999E-05 | -1.3342E-05 | -4.3817E-06 | -1.0949E-06 | 1.71639E-06 | -1.2705E-06 | -4.1592E-06 | -1.0929E-06 | -1.8422E-07 | -2.0689E-06 |
| DLQI1 | -9.2918E-08 | -4.9634E-07 | -4.4113E-06 | 3.15516E-07 | 7.31719E-07 | 9.72248E-07 | -3.5121E-08 | 1.08293E-06 | 9.83537E-07 | -3.9044E-07 | 2.17048E-07 | -4.1999E-05 | 2.94731E-05 | -1.2405E-05 | 9.09454E-07 | 1.90326E-07 | -6.1531E-07 | 1.63529E-08 | -6.8574E-07 | 6.23224E-07 | -5.296E-07 | -1.0931E-06 |
| DLQI2 | 8.66492E-06 | -3.9794E-06 | 3.49661E-06 | -4.5709E-07 | -1.5184E-06 | -2.4386E-07 | -4.6603E-07 | 2.16082E-06 | -6.1592E-07 | 3.42196E-07 | 2.01822E-06 | -1.3342E-05 | -1.2405E-05 | 2.35825E-05 | 4.5466E-07 | -1.6891E-06 | 7.87773E-08 | 1.95432E-08 | -5.7961E-07 | -1.7483E-07 | 3.27259E-07 | 1.10889E-07 |
| DLQI3 | 4.60335E-07 | -9.4326E-08 | -2.1892E-07 | 1.11866E-06 | -4.2958E-08 | -5.5029E-07 | -4.74E-08 | -1.5482E-07 | -1.4928E-07 | 3.46663E-08 | -5.6187E-09 | -4.3817E-06 | 9.09454E-07 | 4.5466E-07 | 2.56387E-06 | -4.9034E-07 | -8.9449E-07 | -2.2895E-07 | -1.0846E-08 | -9.5777E-08 | -3.7704E-08 | -2.8805E-07 |
| DLQI4 | 6.76283E-07 | 4.66334E-07 | 1.08788E-06 | 1.5862E-07 | 1.74595E-06 | -2.3445E-06 | 7.90209E-08 | 8.52675E-08 | -7.345E-07 | 2.10116E-08 | -1.3137E-06 | -1.0949E-06 | 1.90326E-07 | -1.6891E-06 | -4.9034E-07 | 5.24889E-06 | -2.3175E-06 | -1.0992E-07 | -1.4206E-07 | -1.6684E-07 | -1.2736E-07 | -4.0867E-07 |
| DLQI5 | 9.38987E-07 | -1.7369E-07 | -2.7575E-06 | -6.3983E-07 | -7.0481E-07 | 4.42427E-06 | -8.5942E-08 | -1.4495E-06 | 1.53691E-06 | -2.2866E-07 | 1.16966E-06 | 1.71639E-06 | -6.1531E-07 | 7.87773E-08 | -8.9449E-07 | -2.3175E-06 | 5.11821E-06 | -1.1775E-07 | -5.6517E-07 | -3.4755E-07 | -4.7975E-08 | -7.8866E-08 |
| DLQI6 | -2.426E-07 | 2.00807E-07 | -6.0385E-07 | -7.592E-09 | 8.11958E-08 | 4.08429E-07 | -4.8603E-09 | -4.0038E-07 | 3.89446E-07 | -1.2279E-07 | 9.54603E-08 | -1.2705E-06 | 1.63529E-08 | 1.95432E-08 | -2.2895E-07 | -1.0992E-07 | -1.1775E-07 | 1.15623E-06 | -5.9528E-07 | 6.20165E-08 | -3.2895E-07 | 2.43384E-08 |
| DLQI7 | 1.40449E-06 | 7.52623E-07 | 3.75028E-06 | -4.5795E-07 | -1.0072E-06 | -2.3304E-06 | -2.3889E-07 | 2.74108E-06 | -7.4539E-07 | 3.79813E-07 | -1.9882E-06 | -4.1592E-06 | -6.8574E-07 | -5.7961E-07 | -1.0846E-08 | -1.4206E-07 | -5.6517E-07 | -5.9528E-07 | 5.64931E-06 | -5.8607E-07 | -7.5545E-08 | -2.3142E-07 |
| DLQI8 | -3.9619E-07 | 3.1448E-07 | -2.3487E-06 | -2.6406E-08 | 2.27867E-07 | 2.16821E-06 | 9.44947E-08 | -8.0007E-07 | 1.75134E-06 | -6.006E-07 | -4.5729E-08 | -1.0929E-06 | 6.23224E-07 | -1.7483E-07 | -9.5777E-08 | -1.6684E-07 | -3.4755E-07 | 6.20165E-08 | -5.8607E-07 | 3.09417E-06 | -8.4891E-07 | -5.7962E-07 |
| DLQI9 | 1.00112E-06 | -1.54E-07 | 6.88862E-07 | 1.24211E-07 | -2.2064E-07 | -6.5075E-07 | -3.7288E-08 | 2.9714E-07 | -7.2339E-07 | 5.87469E-07 | 1.20064E-07 | -1.8422E-07 | -5.296E-07 | 3.27259E-07 | -3.7704E-08 | -1.2736E-07 | -4.7975E-08 | -3.2895E-07 | -7.5545E-08 | -8.4891E-07 | 1.51288E-06 | -8.132E-08 |
| DLQI10 | 4.5635E-07 | 1.58792E-08 | -2.1259E-07 | -1.3841E-09 | -5.3768E-07 | -8.0133E-08 | -1.4514E-07 | -8.0335E-07 | -2.6193E-07 | 3.69248E-07 | 3.36554E-06 | -2.0689E-06 | -1.0931E-06 | 1.10889E-07 | -2.8805E-07 | -4.0867E-07 | -7.8866E-08 | 2.43384E-08 | -2.3142E-07 | -5.7962E-07 | -8.132E-08 | 4.6967E-06 |

**Blue = Cluster 1; Green = Cluster 2**

DLQI, Dermatology Life Quality Index

**Table D4. Regression Mixture Model 4– DLQI items, age and sex**

**Variance-Covariance Matrix**

| Coefficients | Intercept | DLQI1 | DLQI2 | DLQI3 | DLQI4 | DLQI5 | DLQI6 | DLQI7 | DLQI8 | DLQI9 | DLQI10 | Age | Sex | Intercept | DLQI1 | DLQI2 | DLQI3 | DLQI4 | DLQI5 | DLQI6 | DLQI7 | DLQI8 | DLQI9 | DLQI10 | Age | Sex |
| --- | --- | --- | --- | --- | --- | --- | --- | --- | --- | --- | --- | --- | --- | --- | --- | --- | --- | --- | --- | --- | --- | --- | --- | --- | --- | --- |
| Intercept | 0.000342891 | -4.07287E-05 | -2.18699E-05 | -6.11378E-06 | 2.69283E-06 | -1.62889E-06 | 1.31571E-06 | -1.29155E-06 | -2.03956E-06 | 1.85753E-06 | -4.89634E-06 | -3.17784E-06 | -4.49164E-05 | 1.84609E-05 | 6.49687E-06 | 1.81156E-05 | -4.1999E-06 | 3.04652E-06 | -9.17692E-06 | 8.70215E-07 | 3.99234E-07 | -2.1818E-06 | -2.17769E-06 | -2.25217E-05 | -2.58709E-07 | -5.1016E-06 |
| DLQI1 | -4.07287E-05 | 2.90623E-05 | -1.22068E-05 | 9.14722E-07 | 1.86496E-07 | -5.79045E-07 | 2.54232E-08 | -5.54385E-07 | 4.77315E-07 | -4.26157E-07 | -9.79258E-07 | -3.42011E-08 | 8.38111E-07 | -3.89449E-06 | 1.34837E-06 | -3.66641E-06 | 6.74012E-07 | 4.42366E-07 | 6.77961E-07 | -2.56765E-07 | 1.36464E-06 | 1.01978E-06 | -3.17996E-07 | -1.36373E-06 | 4.93416E-08 | 3.44906E-07 |
| DLQI2 | -2.18699E-05 | -1.22068E-05 | 2.38771E-05 | 5.35388E-07 | -1.89391E-06 | 2.50185E-07 | -7.83225E-08 | -7.71637E-07 | -1.04849E-07 | 1.72191E-07 | 2.95512E-07 | 2.15601E-07 | -3.13997E-06 | 9.05368E-06 | -5.46809E-06 | 1.58842E-06 | -2.89415E-07 | -1.43131E-06 | 5.67755E-07 | -2.75921E-07 | 1.34339E-06 | -5.84759E-07 | 5.06824E-07 | 5.54267E-06 | -3.34222E-08 | -1.20358E-06 |
| DLQI3 | -6.11378E-06 | 9.14722E-07 | 5.35388E-07 | 2.53223E-06 | -5.03896E-07 | -8.66092E-07 | -2.64252E-07 | -5.78717E-09 | -1.08651E-07 | -6.27723E-08 | -2.61803E-07 | 3.34724E-08 | 2.12868E-07 | -1.47346E-06 | 7.88205E-08 | -5.59049E-10 | 1.29477E-06 | -7.41589E-08 | -6.81233E-07 | -6.88183E-08 | -1.39066E-07 | -1.87149E-07 | 2.00697E-08 | -1.72312E-07 | 2.76574E-08 | -9.35819E-08 |
| DLQI4 | 2.69283E-06 | 1.86496E-07 | -1.89391E-06 | -5.03896E-07 | 5.16883E-06 | -2.18953E-06 | -2.65053E-08 | -1.74445E-07 | -1.12962E-07 | -8.70298E-08 | -2.8349E-07 | -6.61787E-08 | -1.01588E-06 | 3.53413E-06 | 2.81781E-07 | 6.64817E-07 | 1.50651E-07 | 1.73289E-06 | -2.02767E-06 | 4.24392E-08 | -6.05868E-10 | -5.61003E-07 | 3.21372E-08 | -1.05053E-06 | -2.86949E-08 | -3.15379E-07 |
| DLQI5 | -1.62889E-06 | -5.79045E-07 | 2.50185E-07 | -8.66092E-07 | -2.18953E-06 | 4.86585E-06 | -1.81705E-07 | -4.91511E-07 | -3.79216E-07 | -9.67674E-08 | -2.60068E-07 | 5.87095E-08 | 8.36444E-07 | -1.50222E-06 | 6.80945E-08 | -2.0137E-06 | -8.34168E-07 | -7.92621E-07 | 3.87115E-06 | -3.71798E-08 | -1.117E-06 | 1.41679E-06 | -3.1824E-07 | 5.80087E-07 | 2.49027E-08 | 4.28556E-07 |
| DLQI6 | 1.31571E-06 | 2.54232E-08 | -7.83225E-08 | -2.64252E-07 | -2.65053E-08 | -1.81705E-07 | 1.17656E-06 | -5.27532E-07 | 2.7887E-08 | -2.89076E-07 | -1.27933E-08 | -4.85402E-08 | -4.5754E-07 | -5.80373E-07 | 9.71931E-08 | -2.24118E-07 | -6.7665E-08 | 1.07989E-07 | 2.27582E-07 | -5.43939E-08 | -2.31929E-07 | 3.98103E-07 | -1.62957E-07 | -1.34271E-07 | 1.78478E-08 | -2.08121E-07 |
| DLQI7 | -1.29155E-06 | -5.54385E-07 | -7.71637E-07 | -5.78717E-09 | -1.74445E-07 | -4.91511E-07 | -5.27532E-07 | 5.5291E-06 | -5.54458E-07 | -2.88374E-08 | -1.40427E-07 | -5.06514E-08 | -9.86069E-07 | 8.74172E-07 | 7.23977E-07 | 3.4115E-06 | -3.43732E-07 | -9.41524E-07 | -1.98964E-06 | -1.18525E-07 | 2.54395E-06 | -8.1788E-07 | 3.36837E-07 | -1.6806E-06 | -2.22778E-08 | 2.41288E-06 |
| DLQI8 | -2.03956E-06 | 4.77315E-07 | -1.04849E-07 | -1.08651E-07 | -1.12962E-07 | -3.79216E-07 | 2.7887E-08 | -5.54458E-07 | 3.02565E-06 | -8.64694E-07 | -6.38048E-07 | 1.43504E-08 | 9.63064E-07 | -6.36651E-07 | 8.06802E-07 | -1.93987E-06 | -7.524E-08 | 1.46985E-07 | 2.06144E-06 | 1.10076E-07 | -7.10799E-07 | 1.78172E-06 | -7.12263E-07 | -8.21657E-07 | -6.32419E-09 | -1.15069E-07 |
| DLQI9 | 1.85753E-06 | -4.26157E-07 | 1.72191E-07 | -6.27723E-08 | -8.70298E-08 | -9.67674E-08 | -2.89076E-07 | -2.88374E-08 | -8.64694E-07 | 1.50871E-06 | -9.93196E-08 | -3.9686E-08 | -5.29038E-07 | -8.36513E-07 | -2.99996E-07 | 8.55704E-07 | 5.32575E-08 | -1.93854E-07 | -8.987E-07 | -4.17525E-08 | 3.17978E-07 | -8.39777E-07 | 6.00884E-07 | 3.23462E-07 | 3.63647E-08 | 7.11666E-07 |
| DLQI10 | -4.89634E-06 | -9.79258E-07 | 2.95512E-07 | -2.61803E-07 | -2.8349E-07 | -2.60068E-07 | -1.27933E-08 | -1.40427E-07 | -6.38048E-07 | -9.93196E-08 | 4.40866E-06 | 5.433E-08 | 2.30636E-08 | -3.44395E-06 | -1.3442E-06 | 4.91343E-07 | -1.55358E-07 | -2.94457E-07 | -8.44822E-07 | -1.14138E-07 | -3.80027E-07 | -7.78694E-07 | 5.98983E-07 | 4.39755E-06 | 7.84146E-08 | 2.00189E-07 |
| Age | -3.17784E-06 | -3.42011E-08 | 2.15601E-07 | 3.34724E-08 | -6.61787E-08 | 5.87095E-08 | -4.85402E-08 | -5.06514E-08 | 1.43504E-08 | -3.9686E-08 | 5.433E-08 | 6.18171E-08 | 3.43494E-07 | -3.88984E-07 | -5.27607E-08 | -2.58109E-07 | 6.69886E-08 | -7.39044E-09 | 1.7604E-07 | 7.50763E-09 | -9.15805E-08 | 1.49574E-09 | 5.16908E-08 | 3.28719E-07 | -3.06175E-09 | 5.00851E-08 |
| Sex | -4.49164E-05 | 8.38111E-07 | -3.13997E-06 | 2.12868E-07 | -1.01588E-06 | 8.36444E-07 | -4.5754E-07 | -9.86069E-07 | 9.63064E-07 | -5.29038E-07 | 2.30636E-08 | 3.43494E-07 | 5.42919E-05 | -8.727E-06 | -1.39258E-07 | -5.4103E-06 | 4.84287E-07 | 4.6557E-07 | 2.59261E-06 | -1.34105E-08 | -1.34785E-06 | 8.86946E-07 | 3.24287E-07 | 2.0327E-06 | 1.50235E-07 | 1.83838E-06 |
| Intercept | 1.84609E-05 | -3.89449E-06 | 9.05368E-06 | -1.47346E-06 | 3.53413E-06 | -1.50222E-06 | -5.80373E-07 | 8.74172E-07 | -6.36651E-07 | -8.36513E-07 | -3.44395E-06 | -3.88984E-07 | -8.727E-06 | 0.005123592 | -0.000510188 | -0.000253888 | -8.10654E-05 | -9.55109E-05 | 3.9268E-06 | -3.17229E-06 | 0.000101351 | -2.17755E-05 | 1.01767E-06 | 2.69106E-05 | -5.2941E-05 | -0.000600459 |
| DLQI1 | 6.49687E-06 | 1.34837E-06 | -5.46809E-06 | 7.88205E-08 | 2.81781E-07 | 6.80945E-08 | 9.71931E-08 | 7.23977E-07 | 8.06802E-07 | -2.99996E-07 | -1.3442E-06 | -5.27607E-08 | -1.39258E-07 | -0.000510188 | 0.000418439 | -0.000102316 | -1.5546E-06 | -1.2449E-05 | -1.00409E-05 | 8.54498E-06 | -7.26172E-05 | 1.17395E-05 | -4.80167E-06 | -3.4539E-05 | -9.14533E-07 | -3.6162E-06 |
| DLQI2 | 1.81156E-05 | -3.66641E-06 | 1.58842E-06 | -5.59049E-10 | 6.64817E-07 | -2.0137E-06 | -2.24118E-07 | 3.4115E-06 | -1.93987E-06 | 8.55704E-07 | 4.91343E-07 | -2.58109E-07 | -5.4103E-06 | -0.000253888 | -0.000102316 | 0.00032371 | 2.6873E-06 | -2.19317E-05 | -5.83941E-05 | 9.48024E-06 | 2.25274E-05 | -2.78684E-05 | 1.09623E-05 | -9.10941E-05 | 1.7629E-06 | 4.88772E-05 |
| DLQI3 | -4.1999E-06 | 6.74012E-07 | -2.89415E-07 | 1.29477E-06 | 1.50651E-07 | -8.34168E-07 | -6.7665E-08 | -3.43732E-07 | -7.524E-08 | 5.32575E-08 | -1.55358E-07 | 6.69886E-08 | 4.84287E-07 | -8.10654E-05 | -1.5546E-06 | 2.6873E-06 | 7.16596E-05 | -8.57032E-07 | -1.82784E-05 | -7.66436E-06 | -1.0911E-05 | -7.62375E-06 | 1.93902E-07 | -8.9932E-06 | 1.08996E-06 | -1.20161E-06 |
| DLQI4 | 3.04652E-06 | 4.42366E-07 | -1.43131E-06 | -7.41589E-08 | 1.73289E-06 | -7.92621E-07 | 1.07989E-07 | -9.41524E-07 | 1.46985E-07 | -1.93854E-07 | -2.94457E-07 | -7.39044E-09 | 4.6557E-07 | -9.55109E-05 | -1.2449E-05 | -2.19317E-05 | -8.57032E-07 | 7.52895E-05 | -6.71327E-06 | -3.04612E-06 | -1.15506E-05 | 2.99653E-08 | 2.79369E-06 | -2.21991E-06 | 1.11051E-06 | -2.20707E-05 |
| DLQI5 | -9.17692E-06 | 6.77961E-07 | 5.67755E-07 | -6.81233E-07 | -2.02767E-06 | 3.87115E-06 | 2.27582E-07 | -1.98964E-06 | 2.06144E-06 | -8.987E-07 | -8.44822E-07 | 1.7604E-07 | 2.59261E-06 | 3.9268E-06 | -1.00409E-05 | -5.83941E-05 | -1.82784E-05 | -6.71327E-06 | 0.000102507 | -4.49405E-06 | -1.41186E-05 | 6.87211E-06 | -4.89844E-06 | 9.2053E-06 | -2.09315E-08 | 1.00421E-05 |
| DLQI6 | 8.70215E-07 | -2.56765E-07 | -2.75921E-07 | -6.88183E-08 | 4.24392E-08 | -3.71798E-08 | -5.43939E-08 | -1.18525E-07 | 1.10076E-07 | -4.17525E-08 | -1.14138E-07 | 7.50763E-09 | -1.34105E-08 | -3.17229E-06 | 8.54498E-06 | 9.48024E-06 | -7.66436E-06 | -3.04612E-06 | -4.49405E-06 | 2.22674E-05 | -1.23169E-05 | -5.26307E-06 | -1.24425E-06 | -3.87224E-06 | -9.9811E-07 | 1.13014E-05 |
| DLQI7 | 3.99234E-07 | 1.36464E-06 | 1.34339E-06 | -1.39066E-07 | -6.05868E-10 | -1.117E-06 | -2.31929E-07 | 2.54395E-06 | -7.10799E-07 | 3.17978E-07 | -3.80027E-07 | -9.15805E-08 | -1.34785E-06 | 0.000101351 | -7.26172E-05 | 2.25274E-05 | -1.0911E-05 | -1.15506E-05 | -1.41186E-05 | -1.23169E-05 | 0.000116234 | -1.59473E-05 | -2.18068E-06 | -2.40112E-05 | -1.05769E-06 | 3.79552E-05 |
| DLQI8 | -2.1818E-06 | 1.01978E-06 | -5.84759E-07 | -1.87149E-07 | -5.61003E-07 | 1.41679E-06 | 3.98103E-07 | -8.1788E-07 | 1.78172E-06 | -8.39777E-07 | -7.78694E-07 | 1.49574E-09 | 8.86946E-07 | -2.17755E-05 | 1.17395E-05 | -2.78684E-05 | -7.62375E-06 | 2.99653E-08 | 6.87211E-06 | -5.26307E-06 | -1.59473E-05 | 5.13236E-05 | -1.43166E-05 | 5.64071E-06 | 9.34078E-07 | -2.48064E-05 |
| DLQI9 | -2.17769E-06 | -3.17996E-07 | 5.06824E-07 | 2.00697E-08 | 3.21372E-08 | -3.1824E-07 | -1.62957E-07 | 3.36837E-07 | -7.12263E-07 | 6.00884E-07 | 5.98983E-07 | 5.16908E-08 | 3.24287E-07 | 1.01767E-06 | -4.80167E-06 | 1.09623E-05 | 1.93902E-07 | 2.79369E-06 | -4.89844E-06 | -1.24425E-06 | -2.18068E-06 | -1.43166E-05 | 2.37917E-05 | -2.46419E-07 | -5.96255E-07 | -1.22958E-05 |
| DLQI10 | -2.25217E-05 | -1.36373E-06 | 5.54267E-06 | -1.72312E-07 | -1.05053E-06 | 5.80087E-07 | -1.34271E-07 | -1.6806E-06 | -8.21657E-07 | 3.23462E-07 | 4.39755E-06 | 3.28719E-07 | 2.0327E-06 | 2.69106E-05 | -3.4539E-05 | -9.10941E-05 | -8.9932E-06 | -2.21991E-06 | 9.2053E-06 | -3.87224E-06 | -2.40112E-05 | 5.64071E-06 | -2.46419E-07 | 0.000163153 | 4.91294E-07 | -3.78967E-05 |
| Age | -2.58709E-07 | 4.93416E-08 | -3.34222E-08 | 2.76574E-08 | -2.86949E-08 | 2.49027E-08 | 1.78478E-08 | -2.22778E-08 | -6.32419E-09 | 3.63647E-08 | 7.84146E-08 | -3.06175E-09 | 1.50235E-07 | -5.2941E-05 | -9.14533E-07 | 1.7629E-06 | 1.08996E-06 | 1.11051E-06 | -2.09315E-08 | -9.9811E-07 | -1.05769E-06 | 9.34078E-07 | -5.96255E-07 | 4.91294E-07 | 1.03182E-06 | 2.91036E-06 |
| Sex | -5.1016E-06 | 3.44906E-07 | -1.20358E-06 | -9.35819E-08 | -3.15379E-07 | 4.28556E-07 | -2.08121E-07 | 2.41288E-06 | -1.15069E-07 | 7.11666E-07 | 2.00189E-07 | 5.00851E-08 | 1.83838E-06 | -0.000600459 | -3.6162E-06 | 4.88772E-05 | -1.20161E-06 | -2.20707E-05 | 1.00421E-05 | 1.13014E-05 | 3.79552E-05 | -2.48064E-05 | -1.22958E-05 | -3.78967E-05 | 2.91036E-06 | 0.000711172 |

**Blue = Cluster 1; Green = Cluster 2**

DLQI, Dermatology Life Quality Index

**Appendix E: Crosswalk from EQ-5D-5L to EQ-5D-3L**

***Defining Outcomes - Source and Target Measures***

The EQ-5D consists of five dimensions (1 = Mobility; 2 = Self-care; 3 = Usual activities; 4 = Pain/Discomfort; and 5 = Anxiety/Depression).[16] Each dimension of the EQ-5D-3L consists of 3 levels (1 = “No problems”; 2 = “Some problems”; and 3 = “Extreme problems”), with health states reported by the patient for the day the questionnaire is administered.[29] There are slight differences in wording between the EQ-5D-5L and -3L versions.[28]

**Results**

***Descriptive Statistics - Characterizing the cohort***

Descriptive statistics are presented in Table E1, and EQ-5D-5L and DLQI scores and the relationship between these measures are presented in Figure 1. The EQ-5D-3L mean score was 0.69 and the median score was 0.74.

**Table E1. Summary of descriptive statistics of the final sample**

| **Descriptive statistics** | |
| --- | --- |
| **Characteristics** | **Estimation Sample (out of 9,759)** |
| EQ-5D-3L score (mean ± SD) | 0.69 ± 0.24 |
| Median: | 0.74 |
| Kurtosis: | 3.27 |
| Skewness: | -1.73 |
| Shapiro Wilk: | p-value < 2.2e^-16^ |

EQ-5D-3L, EQ-5D-3-level; SD, standard deviation

**Fig. 1 EQ-5D-3L and DLQI scores and the relationship between these measures**

**a: Probability distributions for the EQ-5D-3-level (EQ-5D-3L) utility scores and the Dermatology Life Quality Index (DLQI) scores**

**b: Scatter plot of the EQ-5D-3L utility scores and DLQI scores where size of points corresponds to the number of observations**

***Conceptual Overlap***

Spearman’s rank correlation coefficients between the total DLQI score and EQ-5D-3L utility scores and domains are reported in Table E2. Conceptually, EQ-5D and DLQI show moderately negative correlations (EQ-5D-3L crosswalk p = -0.508) between EQ-5D utility scores and DLQI total scores. There was a moderately negative correlation between EQ-5D utility scores and the “Social Activities” DLQI domain (EQ-5D-3L crosswalk = -0.439).

**Table E2. Spearman’s rank correlation coefficient matrix between DLQI and EQ-5D-3L**

|  |  | **EQ-5D-3L** | **Mobility** | **Self-care** | **Usual activities** | **Pain/**  **Discomfort** | **Anxiety/**  **Depression** |
| --- | --- | --- | --- | --- | --- | --- | --- |
| **DLQI** | Total score | -0.508 | 0.362 | 0.405 | 0.444 | 0.414 | 0.392 |
|  | Symptoms | -0.334 | 0.255 | 0.237 | 0.311 | 0.326 | 0.213 |
|  | Feelings | -0.331 | 0.193 | 0.245 | 0.255 | 0.246 | 0.336 |
|  | Daily activities | -0.359 | 0.311 | 0.337 | 0.343 | -0.266 | 0.248 |
|  | Clothing | -0.298 | 0.214 | 0.248 | 0.269 | -0.241 | 0.212 |
|  | Social activities | -0.439 | 0.339 | 0.346 | 0.382 | 0.325 | 0.330 |
|  | Sport | -0.313 | 0.241 | 0.258 | 0.328 | 0.272 | 0.171 |
|  | Work and school | -0.378 | 0.320 | 0.310 | 0.380 | 0.320 | 0.223 |
|  | Personal relationship | -0.325 | 0.255 | 0.292 | 0.310 | 0.225 | 0.226 |
|  | Sex | -0.293 | 0.251 | 0.229 | 0.278 | 0.241 | 0.162 |
|  | Treatment | -0.335 | 0.260 | 0.299 | 0.311 | 0.277 | 0.249 |

DLQI, Dermatology Life Quality Index; EQ-5D-3L, EQ-5D-3-level; EQ-5D-5L

***Model Selection and Performance***

The Akaike Information Criteria (AIC) and Bayesian Information Criteria (BIC) of the models were used to determine the optimal number of clusters based on model fit (Table E3). Coefficients for generating EQ-5D utility scores from DLQI scores for the best model by level/specification are presented in Table E4, including standard errors. Additionally, an app is provided in Online Resource 1 to assist with converting DLQI scores from *de novo* samples to EQ-5D-3L utility scores.

**Table E3. Akaike Information Criteria and Bayesian**

**Information Criteria values by Model and Cluster**

| **Outcome** | | **EQ-5D-3L** | |
| --- | --- | --- | --- |
| **Model** | **Clusters** | **AIC** | **BIC** |
| Model 1 | 1 | -432.59 | -417.24 |
| Model 1 | 2 | -838.23 | -802.42 |
| Model 1 | 3 | -882.46 | -826.18 |
| Model 1 | 4 | -923.55 | -846.80 |
| Model 1 | 5 | -927.29 | -830.08 |
| Model 1 | 6 | -930.14 | -812.46 |
| Model 2 | 1 | -470.83 | -445.25 |
| Model 2 | 2 | -886.31 | -830.03 |
| Model 2 | 3 | -932.89 | -845.92 |
| Model 2 | 4 | -972.72 | -855.04 |
| Model 2 | 5 | -987.74 | -839.36 |
| Model 2 | 6 | -1014.77 | -835.69 |
| Model 3 | 1 | -380.86 | -319.46 |
| Model 3 | 2 | -779.50 | -651.59 |
| Model 3 | 3 | -818.74 | -624.32 |
| Model 3 | 4 | -909.10 | -648.17 |
| Model 3 | 5 | -1000.49 | -673.04 |
| Model 3 | 6 | -862.43 | -534.98 |
| Model 4 | 1 | -395.45 | -323.82 |
| Model 4 | 2 | -803.76 | -655.39 |
| Model 4 | 3 | -842.71 | -617.59 |
| Model 4 | 4 | -880.22 | -578.36 |
| Model 4 | 5 | -954.61 | -575.99 |
| Model 4 | 6 | -879.13 | -500.51 |

AIC, Akaike Information Criteria; BIC, Bayesian Information Criteria

**Table E4. Model coefficients and standard errors**

| **EQ-5D-3L Model Coefficients - Regression Mixture Models** | | | | | | | |
| --- | --- | --- | --- | --- | --- | --- | --- |
|  | | **Cluster 1** | | **Cluster 2** | | | |
| **Level 1 - Total DLQI** | | | | | | | |
| **Cluster Size** | | **n** | **%** | **n** | **%** | | |
|  |  | 1032 | 83.8 | 200 | 16.2 | | |
| **Coefficients** | | **Estimate** | **SE** | **Estimate** | **SE** | | |
| **Intercept** | | 0.863 | 0.006 | 0.663 | 0.039 | | |
| **Total DLQI** | | -0.014 | 0.001 | -0.023 | 0.002 | | |
| **Level 2 - Total DLQI, age and sex** | | | | | | | |
| **Cluster Size** | | **n** | **%** | **n** | **%** | | |
|  |  | 1034 | 83.9 | 198 | 16.1 | | |
| **Coefficients** | | **Estimate** | **SE** | **Estimate** | **SE** | | |
| **Intercept** | | 0.931 | 0.017 | 0.855 | 0.089 | | |
| **Total DLQI** | | -0.014 | 0.001 | -0.024 | 0.002 | | |
| **Age** | | -0.002 | 0.000 | -0.004 | 0.001 | | |
| **Sex** | | 0.017 | 0.009 | -0.021 | 0.041 | | |
| **Level 3 - DLQI Items** | | | | | | | |
| **Cluster Size** | | **n** | **%** | **n** | **%** | | |
|  |  | 1023 | 83.0 | 209 | 17.0 | | |
| **Coefficients** | | **Estimate** | **SE** | **Estimate** | **SE** | | |
| **Intercept** | | 0.978 | 0.017 | 1.115 | 0.057 | | |
| **DLQI Score** | | | | | | | |
| **DLQI Items** |  | **Estimate** | **SE** | **Estimate** | | **SE** | |
|  | **DLQI 1** | -0.036 | 0.007 | -0.062 | | 0.026 | |
|  | **DLQI 2** | -0.038 | 0.006 | -0.049 | | 0.021 | |
|  | **DLQI 3** | 0.001 | 0.002 | -0.006 | | 0.009 | |
|  | **DLQI 4** | 0.006 | 0.003 | -0.013 | | 0.012 | |
|  | **DLQI 5** | -0.007 | 0.003 | -0.029 | | 0.011 | |
|  | **DLQI 6** | 0.000 | 0.001 | -0.004 | | 0.006 | |
|  | **DLQI 7** | -0.019 | 0.003 | -0.062 | | 0.013 | |
|  | **DLQI 8** | 0.001 | 0.002 | 0.001 | | 0.009 | |
|  | **DLQI 9** | -0.002 | 0.002 | -0.016 | | 0.006 | |
|  | **DLQI 10** | 0.001 | 0.003 | -0.021 | | 0.013 | |
| **Level 4 - DLQI Items, age and sex** | | | | | | | |
| **Cluster Size** | | **n** | **%** | **n** | **%** | | |
|  |  | 1031 | 83.7 | 201 | 16.3 | | |
| **Coefficients** | | **Estimate** | **SE** | **Estimate** | **SE** | | |
| **Intercept** | | 1.030 | 0.024 | 1.127 | 0.090 | | |
| **Age** | | -0.001 | 0.000 | 0.000 | 0.001 | | |
| **Sex** | | 0.026 | 0.009 | 0.017 | 0.034 | | |
| **DLQI Score** | | | | | | | |
| **DLQI Items** |  | **Estimate** | **SE** | **Estimate** | | **SE** |  |
|  | **DLQI 1** | -0.035 | 0.007 | -0.063 | | 0.026 |  |
|  | **DLQI 2** | -0.044 | 0.006 | -0.051 | | 0.022 |  |
|  | **DLQI 3** | 0.000 | 0.002 | -0.008 | | 0.010 |  |
|  | **DLQI 4** | 0.006 | 0.003 | -0.013 | | 0.012 |  |
|  | **DLQI 5** | -0.008 | 0.003 | -0.028 | | 0.011 |  |
|  | **DLQI 6** | 0.000 | 0.001 | -0.003 | | 0.006 |  |
|  | **DLQI 7** | -0.018 | 0.003 | -0.062 | | 0.013 |  |
|  | **DLQI 8** | 0.002 | 0.002 | 0.002 | | 0.009 |  |
|  | **DLQI 9** | -0.001 | 0.002 | -0.017 | | 0.007 |  |
|  | **DLQI 10** | 0.000 | 0.003 | -0.020 | | 0.013 |  |

DLQI, Dermatology Life Quality Index; EQ-5D-5L, EQ-5D-5-level, SE, standard error

Note: p values for the mixture model are not provided because they should not be used in the traditional sense, and only in an exploratory manner.[42] P values will be inaccurate because they have not been adjusted for the fact that the same data are used to select the model and compute the p-values.[42]

Regression mixture models were on average the best models for predicting EQ-5D-3L utility scores from DLQI scores (Table E5). The level 2 regression mixture models with total DLQI items and age and sex as independent variables had the lowest MAEs (0.099), and predicted EQ-5D-3L crosswalk values ranging from -0.071 to 0.916. The regression mixture model with individual DLQI items and age and sex as independent variables (level 4) had the lowest RMSE (0.136), and predicted EQ-5D-3L crosswalk values ranging from -0.049 to 0.933.

**Table E5. Model performance for each models and level**

| **Regression type** | **Mean** | **SD** | **Min** | **Max** | **RMSE** | **MAE** | **Rank** |
| --- | --- | --- | --- | --- | --- | --- | --- |
| Observed EQ-5D-3L crosswalk from 5L | 0.693 | 0.236 | -0.594 | 1 | - | - | - |
| **Level 1 Total DLQI** | | | | | | | |
| OLS | 0.693 | 0.121 | 0.248 | 0.835 | 0.203 | 0.143 | 12 |
| Tobit | 0.708 | 0.132 | 0.224 | 0.862 | 0.203 | 0.141 | 11 |
| Two-part: GLM (Logistic) - OLS (Normal) | 0.721 | 0.218 | 0.075 | 1.318 | 0.298 | 0.223 | 17 |
| Two-part: GLM (Logistic) - OLS (Lognormal) | 0.720 | 0.147 | 0.252 | 1 | 0.257 | 0.181 | 13 |
| Two-part: GLM (Logistic) - GLM (Gamma) | 0.821 | 0.151 | -0.472 | 1 | 0.303 | 0.220 | 23 |
| Regression Mixture | 0.716 | 0.163 | -0.030 | 0.863 | 0.142 | 0.100 | 3 |
| **Level 2 Total DLQI + age + sex** | | | | | | | |
| OLS | 0.693 | 0.127 | 0.215 | 0.913 | 0.199 | 0.141 | 9 |
| Tobit | 0.708 | 0.139 | 0.185 | 0.953 | 0.200 | 0.140 | 9 |
| Two-part: GLM (Logistic) - OLS (Normal) | 0.721 | 0.218 | 0.006 | 1.286 | 0.298 | 0.223 | 17 |
| Two-part: GLM (Logistic) - OLS (Lognormal) | 0.720 | 0.147 | 0.204 | 1 | 0.257 | 0.181 | 13 |
| Two-part: GLM (Logistic) - GLM (Gamma) | 0.820 | 0.158 | -0.150 | 1 | 0.311 | 0.228 | 24 |
| Regression Mixture | 0.716 | 0.166 | -0.071 | 0.916 | 0.140 | 0.099 | 1 |
| **Level 3 DLQI Items** | | | | | | | |
| OLS | 0.693 | 0.141 | 0.110 | 0.984 | 0.189 | 0.135 | 6 |
| Tobit | 0.708 | 0.153 | 0.109 | 1.054 | 0.190 | 0.135 | 8 |
| Two-part: GLM (Logistic) - OLS (Normal) | 0.721 | 0.218 | -0.168 | 1.261 | 0.298 | 0.223 | 17 |
| Two-part: GLM (Logistic) - OLS (Lognormal) | 0.720 | 0.147 | 0.111 | 1 | 0.257 | 0.181 | 13 |
| Two-part: GLM (Logistic) - GLM (Gamma) | 0.821 | 0.152 | -0.234 | 1 | 0.302 | 0.218 | 22 |
| Regression Mixture | 0.711 | 0.174 | -0.063 | 0.931 | 0.138 | 0.103 | 3 |
| **Level 4 DLQI Items + age + sex** | | | | | | | |
| OLS | 0.693 | 0.143 | 0.085 | 0.974 | 0.188 | 0.135 | 5 |
| Tobit | 0.708 | 0.155 | 0.083 | 1.042 | 0.189 | 0.135 | 6 |
| Two-part: GLM (Logistic) - OLS (Normal) | 0.721 | 0.218 | -0.167 | 1.318 | 0.298 | 0.223 | 17 |
| Two-part: GLM (Logistic) - OLS (Lognormal) | 0.720 | 0.147 | 0.064 | 1 | 0.257 | 0.181 | 13 |
| Two-part: GLM (Logistic) - GLM (Gamma) | 0.818 | 0.155 | -0.727 | 1 | 0.302 | 0.216 | 21 |
| Regression Mixture | 0.713 | 0.174 | -0.049 | 0.933 | 0.136 | 0.102 | 1 |

DLQI, Dermatology Life Quality Index; EQ-5D-3L, EQ-5D-3-level; GLM, Generalized Linear Model; MAE, mean absolute error; ME, mean error; OLS, Ordinary Least Squares; RMSE, root mean squared error; SD, standard deviation

The proportion of predictions within ±0.05, ±0.10, and ±0.15 of the observed EQ-5D-3L utility scores is reported in Table E6 to provide more information about the predictive ability of the models. Figure 2 demonstrate the predictive ability of the regression mixture model with total DLQI and age and sex as independent variables, where 37.26% of the EQ-5D-3L predictions were within ±0.05 of the observed values, and 60.71% EQ-5D-3Lwere within ±0.10. Figure 2 is a graphical representation of model performance - predicted versus observed EQ-5D utility scores – with element B of the figure showing the range in predicted vs. observed values, and element C showing error distribution.

**Table E6. Summary of the proportion of mapping estimates for each of the EQ-5D-3L models**

| **EQ-5D-3L Crosswalk** |  |  |  |
| --- | --- | --- | --- |
| **Regression type by Data Level** | **±0.05** | **±0.10** | **±0.15** |
| Level 1 Total DLQI | | | |
| OLS | 29.30% | 50.00% | 74.84% |
| Tobit | 29.46% | 50.57% | 77.35% |
| Two-part: GLM (Logistic) - OLS (Normal) | 18.34% | 31.74% | 57.31% |
| Two-part: GLM (Logistic) - OLS (Lognormal) | 23.78% | 42.13% | 64.77% |
| Two-part: GLM (Logistic) - GLM (Gamma) | 16.88% | 31.33% | 60.55% |
| Regression Mixture | 36.69% | 61.53% | 74.84% |
| Level 2 Total DLQI Age and Sex | | | |
| OLS | 27.19% | 50.24% | 77.03% |
| Tobit | 27.52% | 49.76% | 78.33% |
| Two-part: GLM (Logistic) - OLS (Normal) | 14.77% | 28.41% | 52.19% |
| Two-part: GLM (Logistic) - OLS (Lognormal) | 22.73% | 40.34% | 63.64% |
| Two-part: GLM (Logistic) - GLM (Gamma) | 14.77% | 28.98% | 57.87% |
| Regression Mixture | 37.26% | 60.71% | 78.57% |
| Level 3 DLQI Items | | | |
| OLS | 28.17% | 51.22% | 78.81% |
| Tobit | 30.11% | 51.30% | 78.73% |
| Two-part: GLM (Logistic) - OLS (Normal) | 16.96% | 31.09% | 56.09% |
| Two-part: GLM (Logistic) - OLS (Lognormal) | 24.03% | 40.58% | 64.45% |
| Two-part: GLM (Logistic) - GLM (Gamma) | 17.45% | 31.82% | 59.66% |
| Regression Mixture | 33.44% | 57.71% | 75.08% |
| Level 4 DLQI Items Age and Sex | | | |
| OLS | 28.08% | 51.54% | 78.73% |
| Tobit | 28.57% | 50.97% | 80.28% |
| Two-part: GLM (Logistic) - OLS (Normal) | 16.72% | 29.71% | 53.73% |
| Two-part: GLM (Logistic) - OLS (Lognormal) | 23.54% | 41.40% | 64.85% |
| Two-part: GLM (Logistic) - GLM (Gamma) | 16.96% | 34.25% | 61.28% |
| Regression Mixture | 32.63% | 57.71% | 75.97% |

DLQI, Dermatology Life Quality Index; EQ-5D-3L, EQ-5D-3-level; GLM, Generalized Linear Model; OLS, Ordinary

Least Squares

**Fig. 2 Graphical representation of model performance depicting EQ-5D-3L observed versus predicted values**

**a: Range of predictions for observed and predicted EQ-5D-3-level (EQ-5D-3L) utilities**

**b: Scatter plot of the range of predictions for observed versus predicted EQ-5D-3L utility values**

**c: Histogram representing error distribution of the observed versus predicted EQ-5D-3L utility values**

***Uncertainty***

Variance-covariance matrices for EQ-5D-3L were calculated and reported below for all independent variables from the highest ranked model for each level to account for the uncertainty associated with direct mapping, and allow for probabilistic sensitivity analysis in a CUA.[20]

**Table E7. Regression Mixture Model 1 – Total DLQI**

**Variance-Covariance Matrix**

| Coefficients | Intercept | Total DLQI | Intercept | Total DLQI |
| --- | --- | --- | --- | --- |
| Intercept | 4.18117E-05 | -3.63329E-06 | -2.46129E-05 | 1.27533E-06 |
| Total DLQI | -3.63329E-06 | 5.83357E-07 | 4.12096E-06 | -1.85696E-07 |
| Intercept | -2.46129E-05 | 4.12096E-06 | 0.00149399 | -6.25935E-05 |
| TotalDLQI | 1.27533E-06 | -1.85696E-07 | -6.25935E-05 | 5.9907E-06 |

**Blue = Cluster 1; Green = Cluster 2**

DLQI, Dermatology Life Quality Index

**Table E8. Regression Mixture Model 2 – Total DLQI, age and sex**

**Variance-Covariance Matrix**

| Coefficients | Intercept | Total DLQI | Age | Sex | Intercept | Total DLQI | Age | Sex |
| --- | --- | --- | --- | --- | --- | --- | --- | --- |
| Intercept | 0.000292589 | -4.82177E-06 | -4.28381E-06 | -6.51239E-05 | -0.000223308 | 1.63128E-06 | 2.93708E-06 | 3.65617E-05 |
| Total DLQI | -4.82177E-06 | 5.61483E-07 | 2.68303E-08 | 8.94821E-08 | 4.74548E-06 | -1.77216E-07 | 2.13695E-08 | -1.83329E-06 |
| Age | -4.28381E-06 | 2.68303E-08 | 8.17893E-08 | 3.83655E-07 | 3.48386E-06 | 1.45995E-09 | -5.97278E-08 | -1.60091E-07 |
| Sex | -6.51239E-05 | 8.94821E-08 | 3.83655E-07 | 7.23967E-05 | 3.16474E-05 | -6.09615E-07 | -2.11896E-08 | -3.29685E-05 |
| Intercept | -0.000223308 | 4.74548E-06 | 3.48386E-06 | 3.16474E-05 | 0.007896196 | -8.28274E-05 | -0.000103034 | -0.001803165 |
| Total DLQI | 1.63128E-06 | -1.77216E-07 | 1.45995E-09 | -6.09615E-07 | -8.28274E-05 | 5.7677E-06 | 3.43304E-07 | 7.71453E-06 |
| Age | 2.93708E-06 | 2.13695E-08 | -5.97278E-08 | -2.11896E-08 | -0.000103034 | 3.43304E-07 | 1.89689E-06 | 1.02543E-05 |
| Sex | 3.65617E-05 | -1.83329E-06 | -1.60091E-07 | -3.29685E-05 | -0.001803165 | 7.71453E-06 | 1.02543E-05 | 0.001681631 |

**Blue = Cluster 1; Green = Cluster 2**

DLQI, Dermatology Life Quality Index

**Table E9. Regression Mixture Model 3– DLQI items**

**Variance-Covariance Matrix**

| Coefficients | Intercept | DLQI1 | DLQI2 | DLQI3 | DLQI4 | DLQI5 | DLQI6 | DLQI7 | DLQI8 | DLQI9 | DLQI10 | Intercept | DLQI1 | DLQI2 | DLQI3 | DLQI4 | DLQI5 | DLQI6 | DLQI7 | DLQI8 | DLQI9 | DLQI10 |
| --- | --- | --- | --- | --- | --- | --- | --- | --- | --- | --- | --- | --- | --- | --- | --- | --- | --- | --- | --- | --- | --- | --- |
| Intercept | 0.000301161 | -7.44761E-05 | -2.22721E-05 | -7.9832E-06 | -4.44384E-06 | 4.30898E-06 | -2.38769E-06 | -7.27537E-06 | -6.6066E-07 | -7.558E-07 | -3.65201E-06 | -0.000146 | 2.56381E-05 | 6.74068E-06 | 4.42364E-06 | 3.37229E-06 | 6.27742E-06 | 5.21649E-07 | -6.5915E-06 | 1.05145E-06 | 1.67308E-06 | -4.70719E-06 |
| DLQI1 | -7.44761E-05 | 5.09E-05 | -1.91633E-05 | 1.59629E-06 | 3.68236E-07 | -1.48016E-06 | 2.73325E-08 | -1.8287E-06 | 4.94882E-07 | -7.31457E-07 | -1.41897E-06 | 2.2096E-05 | -2.00452E-05 | -2.32869E-06 | -1.81239E-07 | 2.63486E-06 | 1.10859E-06 | 5.53095E-07 | 5.38605E-06 | -1.03349E-06 | -7.40868E-07 | 3.63639E-06 |
| DLQI2 | -2.22721E-05 | -1.91633E-05 | 3.71246E-05 | 8.05278E-07 | -2.30618E-06 | -2.81291E-07 | 6.82557E-08 | -1.52537E-06 | -7.49661E-08 | 3.10929E-07 | -8.30684E-07 | 1.89308E-05 | -1.22972E-06 | -8.72106E-06 | -1.65427E-07 | -2.7647E-06 | 1.01817E-06 | -1.29965E-06 | 1.59816E-06 | 8.9697E-07 | -9.78354E-07 | 5.50282E-06 |
| DLQI3 | -7.9832E-06 | 1.59629E-06 | 8.05278E-07 | 4.6743E-06 | -7.55523E-07 | -1.81701E-06 | -3.2778E-07 | 3.46806E-08 | -1.89865E-07 | -8.45013E-08 | -4.57574E-07 | 3.57972E-06 | -6.06934E-07 | 5.04167E-07 | -2.51778E-07 | -2.88665E-07 | -8.47558E-07 | 1.25362E-07 | 3.90128E-07 | -2.94047E-07 | 2.10535E-07 | -3.8427E-08 |
| DLQI4 | -4.44384E-06 | 3.68236E-07 | -2.30618E-06 | -7.55523E-07 | 8.51877E-06 | -3.62833E-06 | -1.83678E-07 | -1.0665E-07 | -1.91488E-07 | -8.42639E-08 | -3.73247E-07 | 2.1732E-06 | 8.99618E-07 | -1.04338E-06 | 1.48036E-09 | 3.3153E-06 | -2.61025E-06 | 4.46989E-07 | -3.32839E-07 | -1.06864E-06 | 9.13081E-07 | -3.22757E-07 |
| DLQI5 | 4.30898E-06 | -1.48016E-06 | -2.81291E-07 | -1.81701E-06 | -3.62833E-06 | 8.69662E-06 | -3.26778E-07 | -4.87068E-07 | -1.0511E-06 | 2.82004E-08 | -3.62719E-07 | 2.78284E-07 | 1.10832E-06 | 1.18219E-06 | -7.86823E-07 | -2.14212E-06 | 2.92006E-06 | 1.7334E-08 | -8.35033E-07 | 1.09995E-06 | -4.85951E-07 | -1.33356E-06 |
| DLQI6 | -2.38769E-06 | 2.73325E-08 | 6.82557E-08 | -3.2778E-07 | -1.83678E-07 | -3.26778E-07 | 2.01896E-06 | -8.87831E-07 | -1.83508E-08 | -5.56503E-07 | 9.35264E-08 | 8.13107E-07 | 9.90503E-07 | -6.27247E-07 | 8.48772E-08 | 1.30485E-07 | 2.44255E-08 | -8.27933E-07 | 1.28691E-07 | 5.17442E-07 | 8.72194E-08 | -1.91548E-07 |
| DLQI7 | -7.27537E-06 | -1.8287E-06 | -1.52537E-06 | 3.46806E-08 | -1.0665E-07 | -4.87068E-07 | -8.87831E-07 | 9.46405E-06 | -8.4409E-07 | -1.41192E-07 | -2.12589E-07 | 4.27122E-06 | 4.6299E-06 | 4.9294E-06 | -4.29269E-07 | -4.94063E-07 | -2.5778E-06 | 8.04154E-07 | -3.69698E-07 | -1.00143E-06 | 1.01426E-06 | -3.61871E-06 |
| DLQI8 | -6.6066E-07 | 4.94882E-07 | -7.49661E-08 | -1.89865E-07 | -1.91488E-07 | -1.0511E-06 | -1.83508E-08 | -8.4409E-07 | 4.7611E-06 | -1.24734E-06 | -7.9195E-07 | -9.51598E-07 | -9.00648E-07 | -4.36227E-07 | -2.16282E-07 | -2.63836E-07 | 1.17726E-06 | 2.73179E-07 | 1.20816E-07 | 3.32505E-07 | -5.48431E-07 | -6.10154E-08 |
| DLQI9 | -7.558E-07 | -7.31457E-07 | 3.10929E-07 | -8.45013E-08 | -8.42639E-08 | 2.82004E-08 | -5.56503E-07 | -1.41192E-07 | -1.24734E-06 | 2.42628E-06 | -3.16481E-07 | 3.01248E-06 | 5.38177E-08 | 5.96616E-07 | 1.81122E-07 | 1.49724E-07 | -6.65781E-07 | 2.68031E-07 | -5.99311E-08 | -6.21866E-07 | 1.57634E-07 | -2.70289E-07 |
| DLQI10 | -3.65201E-06 | -1.41897E-06 | -8.30684E-07 | -4.57574E-07 | -3.73247E-07 | -3.62719E-07 | 9.35264E-08 | -2.12589E-07 | -7.9195E-07 | -3.16481E-07 | 7.3533E-06 | -3.80831E-08 | 2.56481E-06 | 3.11998E-06 | -2.37046E-07 | -5.09665E-07 | -1.61748E-06 | -2.50896E-09 | -1.1607E-06 | -3.3502E-07 | 5.43381E-07 | -2.18041E-07 |
| Intercept | -0.000146 | 2.2096E-05 | 1.89308E-05 | 3.57972E-06 | 2.1732E-06 | 2.78284E-07 | 8.13107E-07 | 4.27122E-06 | -9.51598E-07 | 3.01248E-06 | -3.80831E-08 | 0.003218696 | -0.000843392 | -0.000194129 | -4.7202E-05 | -8.41135E-05 | 7.44955E-06 | -6.30856E-05 | 9.50165E-05 | 1.9523E-05 | -6.59205E-05 | 4.47151E-05 |
| DLQI1 | 2.56381E-05 | -2.00452E-05 | -1.22972E-06 | -6.06934E-07 | 8.99618E-07 | 1.10832E-06 | 9.90503E-07 | 4.6299E-06 | -9.00648E-07 | 5.38177E-08 | 2.56481E-06 | -0.000843392 | 0.000671304 | -0.000156892 | -4.96883E-06 | -2.57638E-05 | -2.05711E-05 | 8.54484E-06 | -0.000114916 | 1.90523E-05 | 6.67327E-06 | -5.92592E-05 |
| DLQI2 | 6.74068E-06 | -2.32869E-06 | -8.72106E-06 | 5.04167E-07 | -1.04338E-06 | 1.18219E-06 | -6.27247E-07 | 4.9294E-06 | -4.36227E-07 | 5.96616E-07 | 3.11998E-06 | -0.000194129 | -0.000156892 | 0.000455419 | 5.37093E-06 | -4.94238E-05 | -4.52879E-05 | 2.24022E-05 | -8.49939E-06 | -2.34089E-05 | 1.52016E-05 | -9.06515E-05 |
| DLQI3 | 4.42364E-06 | -1.81239E-07 | -1.65427E-07 | -2.51778E-07 | 1.48036E-09 | -7.86823E-07 | 8.48772E-08 | -4.29269E-07 | -2.16282E-07 | 1.81122E-07 | -2.37046E-07 | -4.7202E-05 | -4.96883E-06 | 5.37093E-06 | 8.49255E-05 | -5.92374E-06 | -1.88563E-05 | -1.21431E-05 | -1.54312E-06 | -1.56358E-05 | 1.2932E-06 | -1.11059E-05 |
| DLQI4 | 3.37229E-06 | 2.63486E-06 | -2.7647E-06 | -2.88665E-07 | 3.3153E-06 | -2.14212E-06 | 1.30485E-07 | -4.94063E-07 | -2.63836E-07 | 1.49724E-07 | -5.09665E-07 | -8.41135E-05 | -2.57638E-05 | -4.94238E-05 | -5.92374E-06 | 0.000135262 | -1.59656E-05 | -9.01E-07 | -5.73016E-06 | -9.95035E-06 | 1.11545E-05 | -2.26768E-06 |
| DLQI5 | 6.27742E-06 | 1.10859E-06 | 1.01817E-06 | -8.47558E-07 | -2.61025E-06 | 2.92006E-06 | 2.44255E-08 | -2.5778E-06 | 1.17726E-06 | -6.65781E-07 | -1.61748E-06 | 7.44955E-06 | -2.05711E-05 | -4.52879E-05 | -1.88563E-05 | -1.59656E-05 | 0.000121595 | -6.80673E-06 | -7.66634E-06 | -3.02774E-06 | -1.00652E-05 | -3.90327E-06 |
| DLQI6 | 5.21649E-07 | 5.53095E-07 | -1.29965E-06 | 1.25362E-07 | 4.46989E-07 | 1.7334E-08 | -8.27933E-07 | 8.04154E-07 | 2.73179E-07 | 2.68031E-07 | -2.50896E-09 | -6.30856E-05 | 8.54484E-06 | 2.24022E-05 | -1.21431E-05 | -9.01E-07 | -6.80673E-06 | 3.61105E-05 | -1.90661E-05 | -8.62368E-06 | -1.44568E-06 | -9.65025E-06 |
| DLQI7 | -6.5915E-06 | 5.38605E-06 | 1.59816E-06 | 3.90128E-07 | -3.32839E-07 | -8.35033E-07 | 1.28691E-07 | -3.69698E-07 | 1.20816E-07 | -5.99311E-08 | -1.1607E-06 | 9.50165E-05 | -0.000114916 | -8.49939E-06 | -1.54312E-06 | -5.73016E-06 | -7.66634E-06 | -1.90661E-05 | 0.000161532 | -2.02231E-05 | -7.2288E-06 | -6.37026E-06 |
| DLQI8 | 1.05145E-06 | -1.03349E-06 | 8.9697E-07 | -2.94047E-07 | -1.06864E-06 | 1.09995E-06 | 5.17442E-07 | -1.00143E-06 | 3.32505E-07 | -6.21866E-07 | -3.3502E-07 | 1.9523E-05 | 1.90523E-05 | -2.34089E-05 | -1.56358E-05 | -9.95035E-06 | -3.02774E-06 | -8.62368E-06 | -2.02231E-05 | 7.70373E-05 | -2.39621E-05 | 3.58446E-06 |
| DLQI9 | 1.67308E-06 | -7.40868E-07 | -9.78354E-07 | 2.10535E-07 | 9.13081E-07 | -4.85951E-07 | 8.72194E-08 | 1.01426E-06 | -5.48431E-07 | 1.57634E-07 | 5.43381E-07 | -6.59205E-05 | 6.67327E-06 | 1.52016E-05 | 1.2932E-06 | 1.11545E-05 | -1.00652E-05 | -1.44568E-06 | -7.2288E-06 | -2.39621E-05 | 4.04475E-05 | -9.07113E-06 |
| DLQI10 | -4.70719E-06 | 3.63639E-06 | 5.50282E-06 | -3.8427E-08 | -3.22757E-07 | -1.33356E-06 | -1.91548E-07 | -3.61871E-06 | -6.10154E-08 | -2.70289E-07 | -2.18041E-07 | 4.47151E-05 | -5.92592E-05 | -9.06515E-05 | -1.11059E-05 | -2.26768E-06 | -3.90327E-06 | -9.65025E-06 | -6.37026E-06 | 3.58446E-06 | -9.07113E-06 | 0.000180285 |

**Blue = Cluster 1; Green = Cluster 2**

DLQI, Dermatology Life Quality Index

**Table E10. Regression Mixture Model 4– DLQI items, age and sex**

**Variance-Covariance Matrix**

| Coefficients | Intercept | DLQI1 | DLQI2 | DLQI3 | DLQI4 | DLQI5 | DLQI6 | DLQI7 | DLQI8 | DLQI9 | DLQI10 | Age | Sex | Intercept | DLQI1 | DLQI2 | DLQI3 | DLQI4 | DLQI5 | DLQI6 | DLQI7 | DLQI8 | DLQI9 | DLQI10 | Age | Sex |
| --- | --- | --- | --- | --- | --- | --- | --- | --- | --- | --- | --- | --- | --- | --- | --- | --- | --- | --- | --- | --- | --- | --- | --- | --- | --- | --- |
| Intercept | 0.000560618 | -6.83695E-05 | -3.41306E-05 | -1.00285E-05 | -4.49027E-07 | 3.0834E-07 | 1.71183E-06 | -2.42695E-06 | -2.9627E-06 | 3.53955E-06 | -6.48014E-06 | -4.90809E-06 | -7.15888E-05 | -0.000219951 | 1.90429E-05 | 2.0113E-05 | 8.70459E-07 | 4.23578E-06 | 1.60908E-06 | -5.62717E-07 | -7.3961E-06 | 3.26215E-06 | -6.77916E-08 | -1.21354E-05 | 2.07697E-06 | 2.50594E-05 |
| DLQI1 | -6.83695E-05 | 4.79273E-05 | -1.83284E-05 | 1.39987E-06 | 5.34031E-07 | -1.38515E-06 | 2.12909E-07 | -1.35987E-06 | 2.4664E-07 | -6.20964E-07 | -1.11413E-06 | -6.00011E-08 | -3.23107E-08 | 7.1334E-06 | -1.54767E-05 | -1.66408E-06 | 4.53611E-07 | 2.95572E-06 | -7.53217E-07 | 2.11914E-07 | 5.50503E-06 | -1.70458E-06 | 2.93128E-07 | 2.04352E-06 | 2.64652E-07 | -6.80497E-07 |
| DLQI2 | -3.41306E-05 | -1.83284E-05 | 3.66109E-05 | 9.63442E-07 | -2.5965E-06 | 1.61442E-07 | -2.11919E-07 | -1.6896E-06 | 7.54605E-08 | 1.10981E-07 | -7.93056E-07 | 2.9698E-07 | -2.88586E-06 | 3.60441E-05 | -2.85672E-06 | -8.50552E-06 | -1.37817E-07 | -2.9882E-06 | 2.05022E-06 | -8.53593E-07 | 1.72711E-06 | 9.98487E-07 | -1.42972E-06 | 5.62725E-06 | -3.19217E-07 | -3.92071E-06 |
| DLQI3 | -1.00285E-05 | 1.39987E-06 | 9.63442E-07 | 4.37675E-06 | -7.35246E-07 | -1.64258E-06 | -3.63451E-07 | -5.02917E-08 | -1.79542E-07 | -1.33606E-07 | -3.36991E-07 | 4.71945E-08 | 4.68792E-07 | 1.55471E-06 | -4.05049E-07 | 3.64579E-07 | 2.63447E-07 | -3.48837E-07 | -9.73662E-07 | 5.07902E-08 | 2.46484E-07 | -3.74217E-07 | 1.66654E-07 | 1.64776E-07 | 3.07017E-08 | -6.59538E-07 |
| DLQI4 | -4.49027E-07 | 5.34031E-07 | -2.5965E-06 | -7.35246E-07 | 8.38044E-06 | -3.6859E-06 | -8.42755E-08 | 1.47446E-08 | -2.86846E-07 | -3.78249E-11 | -3.83187E-07 | -7.33875E-08 | -7.30978E-07 | 1.17896E-06 | 1.19064E-06 | -4.3728E-07 | 6.20907E-09 | 3.55604E-06 | -3.3799E-06 | 3.98276E-07 | -1.48405E-07 | -1.15088E-06 | 1.05752E-06 | -7.02E-07 | 4.03012E-08 | -9.72228E-07 |
| DLQI5 | 3.0834E-07 | -1.38515E-06 | 1.61442E-07 | -1.64258E-06 | -3.6859E-06 | 8.44477E-06 | -3.67493E-07 | -5.9157E-07 | -9.471E-07 | -2.70054E-08 | -3.46628E-07 | 7.5376E-08 | -1.45432E-07 | 2.77303E-06 | 3.24568E-07 | 1.0096E-06 | -1.16581E-06 | -2.71008E-06 | 3.66025E-06 | 1.34272E-07 | -5.47172E-07 | 1.10502E-06 | -7.04983E-07 | -8.34627E-07 | -6.22776E-08 | 1.71139E-06 |
| DLQI6 | 1.71183E-06 | 2.12909E-07 | -2.11919E-07 | -3.63451E-07 | -8.42755E-08 | -3.67493E-07 | 1.99267E-06 | -7.78378E-07 | -8.25882E-08 | -4.63693E-07 | 6.08895E-08 | -8.27069E-08 | -6.38235E-07 | -4.81944E-07 | 6.70843E-07 | -2.18304E-07 | 6.6348E-08 | 2.61476E-07 | -1.11889E-07 | -8.07781E-07 | 7.22894E-08 | 6.04572E-07 | 2.03399E-08 | -3.52642E-07 | 5.40268E-08 | -9.46186E-07 |
| DLQI7 | -2.42695E-06 | -1.35987E-06 | -1.6896E-06 | -5.02917E-08 | 1.47446E-08 | -5.9157E-07 | -7.78378E-07 | 8.95867E-06 | -8.55195E-07 | -4.72754E-08 | -3.04449E-07 | -8.49856E-08 | -1.38361E-06 | 3.90946E-06 | 4.5231E-06 | 4.39621E-06 | -3.4376E-07 | -6.35583E-07 | -2.25057E-06 | 7.25058E-07 | -1.13443E-07 | -1.01294E-06 | 7.60828E-07 | -3.55133E-06 | -1.33341E-08 | 1.11728E-06 |
| DLQI8 | -2.9627E-06 | 2.4664E-07 | 7.54605E-08 | -1.79542E-07 | -2.86846E-07 | -9.471E-07 | -8.25882E-08 | -8.55195E-07 | 4.66127E-06 | -1.22492E-06 | -6.96186E-07 | 3.85866E-08 | 1.60941E-06 | 2.34281E-06 | -1.13534E-06 | -5.84424E-07 | -4.44335E-07 | -3.84736E-07 | 1.34968E-06 | 4.19047E-07 | 2.29375E-07 | 2.58717E-07 | -6.11529E-07 | 2.81406E-07 | -7.68125E-08 | 7.15798E-07 |
| DLQI9 | 3.53955E-06 | -6.20964E-07 | 1.10981E-07 | -1.33606E-07 | -3.78249E-11 | -2.70054E-08 | -4.63693E-07 | -4.72754E-08 | -1.22492E-06 | 2.38209E-06 | -3.79034E-07 | -7.68526E-08 | -1.31138E-06 | 1.05725E-06 | 6.18826E-07 | 6.14488E-07 | 1.60251E-07 | 4.54377E-08 | -8.20977E-07 | 2.34441E-07 | -1.2165E-07 | -7.18966E-07 | 2.86631E-07 | -5.82431E-07 | 3.2595E-08 | 1.05131E-06 |
| DLQI10 | -6.48014E-06 | -1.11413E-06 | -7.93056E-07 | -3.36991E-07 | -3.83187E-07 | -3.46628E-07 | 6.08895E-08 | -3.04449E-07 | -6.96186E-07 | -3.79034E-07 | 6.99206E-06 | 5.55274E-08 | 2.04775E-07 | -2.41598E-07 | 2.18311E-06 | 2.75447E-06 | 1.07566E-08 | -5.41208E-07 | -1.17539E-06 | -5.97262E-08 | -1.08918E-06 | -2.29628E-07 | 3.67626E-07 | -3.98482E-07 | -1.1453E-08 | 9.30786E-07 |
| Age | -4.90809E-06 | -6.00011E-08 | 2.9698E-07 | 4.71945E-08 | -7.33875E-08 | 7.5376E-08 | -8.27069E-08 | -8.49856E-08 | 3.85866E-08 | -7.68526E-08 | 5.55274E-08 | 9.75544E-08 | 5.24727E-07 | 1.62698E-06 | 7.12764E-08 | -2.45296E-07 | 3.95684E-08 | -4.31353E-08 | 1.37881E-07 | 4.44476E-08 | -5.0478E-09 | -4.89607E-08 | 2.33029E-08 | 1.67707E-07 | -4.8494E-08 | -1.08612E-07 |
| Sex | -7.15888E-05 | -3.23107E-08 | -2.88586E-06 | 4.68792E-07 | -7.30978E-07 | -1.45432E-07 | -6.38235E-07 | -1.38361E-06 | 1.60941E-06 | -1.31138E-06 | 2.04775E-07 | 5.24727E-07 | 8.88955E-05 | 2.66632E-05 | -1.32495E-06 | -6.65509E-06 | -4.39909E-07 | 2.0738E-06 | -3.07476E-07 | -1.28098E-06 | -1.74251E-06 | 2.4776E-06 | 4.38701E-07 | 4.50831E-06 | -1.56027E-07 | -2.81436E-05 |
| Intercept | -0.000219951 | 7.1334E-06 | 3.60441E-05 | 1.55471E-06 | 1.17896E-06 | 2.77303E-06 | -4.81944E-07 | 3.90946E-06 | 2.34281E-06 | 1.05725E-06 | -2.41598E-07 | 1.62698E-06 | 2.66632E-05 | 0.008108344 | -0.000731049 | -0.000424081 | -9.92683E-05 | -0.00015017 | -1.92026E-05 | -4.60266E-06 | 0.000110863 | 1.01433E-05 | -8.07416E-06 | 0.000101823 | -8.45843E-05 | -0.001031827 |
| DLQI1 | 1.90429E-05 | -1.54767E-05 | -2.85672E-06 | -4.05049E-07 | 1.19064E-06 | 3.24568E-07 | 6.70843E-07 | 4.5231E-06 | -1.13534E-06 | 6.18826E-07 | 2.18311E-06 | 7.12764E-08 | -1.32495E-06 | -0.000731049 | 0.000671297 | -0.000160841 | -6.55958E-06 | -2.29763E-05 | -1.84302E-05 | 9.56275E-06 | -0.000118034 | 1.84688E-05 | 8.29735E-06 | -5.33599E-05 | -2.33365E-06 | -2.1113E-05 |
| DLQI2 | 2.0113E-05 | -1.66408E-06 | -8.50552E-06 | 3.64579E-07 | -4.3728E-07 | 1.0096E-06 | -2.18304E-07 | 4.39621E-06 | -5.84424E-07 | 6.14488E-07 | 2.75447E-06 | -2.45296E-07 | -6.65509E-06 | -0.000424081 | -0.000160841 | 0.000464249 | 9.33065E-06 | -4.50063E-05 | -4.47212E-05 | 2.01948E-05 | -1.04134E-05 | -2.34926E-05 | 1.3767E-05 | -9.68322E-05 | 4.07028E-06 | 4.69833E-05 |
| DLQI3 | 8.70459E-07 | 4.53611E-07 | -1.37817E-07 | 2.63447E-07 | 6.20907E-09 | -1.16581E-06 | 6.6348E-08 | -3.4376E-07 | -4.44335E-07 | 1.60251E-07 | 1.07566E-08 | 3.95684E-08 | -4.39909E-07 | -9.92683E-05 | -6.55958E-06 | 9.33065E-06 | 9.05169E-05 | -4.57784E-06 | -2.53142E-05 | -1.34183E-05 | -4.22377E-07 | -1.61899E-05 | 2.33258E-06 | -1.28928E-05 | 1.25482E-06 | -1.32117E-05 |
| DLQI4 | 4.23578E-06 | 2.95572E-06 | -2.9882E-06 | -3.48837E-07 | 3.55604E-06 | -2.71008E-06 | 2.61476E-07 | -6.35583E-07 | -3.84736E-07 | 4.54377E-08 | -5.41208E-07 | -4.31353E-08 | 2.0738E-06 | -0.00015017 | -2.29763E-05 | -4.50063E-05 | -4.57784E-06 | 0.00013691 | -1.97518E-05 | -4.64348E-06 | -9.33507E-06 | -5.17301E-06 | 1.00119E-05 | -1.49571E-06 | 1.85818E-06 | -5.29533E-05 |
| DLQI5 | 1.60908E-06 | -7.53217E-07 | 2.05022E-06 | -9.73662E-07 | -3.3799E-06 | 3.66025E-06 | -1.11889E-07 | -2.25057E-06 | 1.34968E-06 | -8.20977E-07 | -1.17539E-06 | 1.37881E-07 | -3.07476E-07 | -1.92026E-05 | -1.84302E-05 | -4.47212E-05 | -2.53142E-05 | -1.97518E-05 | 0.000126859 | -5.02766E-06 | -6.41024E-06 | -2.56893E-06 | -1.19017E-05 | -2.88453E-06 | -2.06275E-07 | 5.49891E-05 |
| DLQI6 | -5.62717E-07 | 2.11914E-07 | -8.53593E-07 | 5.07902E-08 | 3.98276E-07 | 1.34272E-07 | -8.07781E-07 | 7.25058E-07 | 4.19047E-07 | 2.34441E-07 | -5.97262E-08 | 4.44476E-08 | -1.28098E-06 | -4.60266E-06 | 9.56275E-06 | 2.01948E-05 | -1.34183E-05 | -4.64348E-06 | -5.02766E-06 | 3.89331E-05 | -1.82028E-05 | -1.11084E-05 | -9.12001E-07 | -9.96414E-06 | -1.39602E-06 | 2.03523E-05 |
| DLQI7 | -7.3961E-06 | 5.50503E-06 | 1.72711E-06 | 2.46484E-07 | -1.48405E-07 | -5.47172E-07 | 7.22894E-08 | -1.13443E-07 | 2.29375E-07 | -1.2165E-07 | -1.08918E-06 | -5.0478E-09 | -1.74251E-06 | 0.000110863 | -0.000118034 | -1.04134E-05 | -4.22377E-07 | -9.33507E-06 | -6.41024E-06 | -1.82028E-05 | 0.000170364 | -2.44359E-05 | -7.35179E-06 | -9.36243E-06 | -4.83328E-07 | 3.52696E-05 |
| DLQI8 | 3.26215E-06 | -1.70458E-06 | 9.98487E-07 | -3.74217E-07 | -1.15088E-06 | 1.10502E-06 | 6.04572E-07 | -1.01294E-06 | 2.58717E-07 | -7.18966E-07 | -2.29628E-07 | -4.89607E-08 | 2.4776E-06 | 1.01433E-05 | 1.84688E-05 | -2.34926E-05 | -1.61899E-05 | -5.17301E-06 | -2.56893E-06 | -1.11084E-05 | -2.44359E-05 | 8.01503E-05 | -2.52813E-05 | 7.68918E-06 | 5.90144E-07 | -4.20698E-05 |
| DLQI9 | -6.77916E-08 | 2.93128E-07 | -1.42972E-06 | 1.66654E-07 | 1.05752E-06 | -7.04983E-07 | 2.03399E-08 | 7.60828E-07 | -6.11529E-07 | 2.86631E-07 | 3.67626E-07 | 2.33029E-08 | 4.38701E-07 | -8.07416E-06 | 8.29735E-06 | 1.3767E-05 | 2.33258E-06 | 1.00119E-05 | -1.19017E-05 | -9.12001E-07 | -7.35179E-06 | -2.52813E-05 | 4.23878E-05 | -8.79214E-06 | -9.73872E-07 | -1.93428E-05 |
| DLQI10 | -1.21354E-05 | 2.04352E-06 | 5.62725E-06 | 1.64776E-07 | -7.02E-07 | -8.34627E-07 | -3.52642E-07 | -3.55133E-06 | 2.81406E-07 | -5.82431E-07 | -3.98482E-07 | 1.67707E-07 | 4.50831E-06 | 0.000101823 | -5.33599E-05 | -9.68322E-05 | -1.28928E-05 | -1.49571E-06 | -2.88453E-06 | -9.96414E-06 | -9.36243E-06 | 7.68918E-06 | -8.79214E-06 | 0.000178676 | -9.32352E-07 | -2.07822E-05 |
| Age | 2.07697E-06 | 2.64652E-07 | -3.19217E-07 | 3.07017E-08 | 4.03012E-08 | -6.22776E-08 | 5.40268E-08 | -1.33341E-08 | -7.68125E-08 | 3.2595E-08 | -1.1453E-08 | -4.8494E-08 | -1.56027E-07 | -8.45843E-05 | -2.33365E-06 | 4.07028E-06 | 1.25482E-06 | 1.85818E-06 | -2.06275E-07 | -1.39602E-06 | -4.83328E-07 | 5.90144E-07 | -9.73872E-07 | -9.32352E-07 | 1.63105E-06 | 6.13188E-06 |
| Sex | 2.50594E-05 | -6.80497E-07 | -3.92071E-06 | -6.59538E-07 | -9.72228E-07 | 1.71139E-06 | -9.46186E-07 | 1.11728E-06 | 7.15798E-07 | 1.05131E-06 | 9.30786E-07 | -1.08612E-07 | -2.81436E-05 | -0.001031827 | -2.1113E-05 | 4.69833E-05 | -1.32117E-05 | -5.29533E-05 | 5.49891E-05 | 2.03523E-05 | 3.52696E-05 | -4.20698E-05 | -1.93428E-05 | -2.07822E-05 | 6.13188E-06 | 0.001172362 |

**Blue = Cluster 1; Green = Cluster 2**

DLQI, Dermatology Life Quality Index
